# A machine learning approach to leveraging electronic health records for enhanced omics analysis

**DOI:** 10.1038/s42256-024-00974-9

**Published:** 2025-01-16

**Authors:** Samson J. Mataraso, Camilo A. Espinosa, David Seong, S. Momsen Reincke, Eloise Berson, Jonathan D. Reiss, Yeasul Kim, Marc Ghanem, Chi-Hung Shu, Tomin James, Yuqi Tan, Sayane Shome, Ina A. Stelzer, Dorien Feyaerts, Ronald J. Wong, Gary M. Shaw, Martin S. Angst, Brice Gaudilliere, David K. Stevenson, Nima Aghaeepour

**Affiliations:** 1 Department of Anesthesiology, Perioperative and Pain Medicine, Stanford University School of Medicine, Stanford, CA USA; 2 Department of Pediatrics, Stanford University School of Medicine, Stanford, CA USA; 3 Department of Biomedical Data Science, Stanford University School of Medicine, Stanford, CA USA; 4 Immunology Program, Stanford University School of Medicine, Stanford, CA USA; 5 Medical Scientist Training Program, Stanford University School of Medicine, Stanford, CA USA; 6 Department of Pathology, Stanford University School of Medicine, Stanford, CA USA; 7 Department of Microbiology and Immunology, Stanford University School of Medicine, Stanford, CA USA; 8 Department of Pathology, University of California San Diego, La Jolla, CA USA

**Keywords:** Machine learning, Cancer epidemiology, Data integration, Pregnancy outcome

## Abstract

Omics studies produce a large number of measurements, enabling the development, validation and interpretation of systems-level biological models. Large cohorts are required to power these complex models; yet, the cohort size remains limited due to clinical and budgetary constraints. We introduce clinical and omics multimodal analysis enhanced with transfer learning (COMET), a machine learning framework that incorporates large, observational electronic health record databases and transfer learning to improve the analysis of small datasets from omics studies. By pretraining on electronic health record data and adaptively blending both early and late fusion strategies, COMET overcomes the limitations of existing multimodal machine learning methods. Using two independent datasets, we showed that COMET improved the predictive modelling performance and biological discovery compared with the analysis of omics data with traditional methods. By incorporating electronic health record data into omics analyses, COMET enables more precise patient classifications, beyond the simplistic binary reduction to cases and controls. This framework can be broadly applied to the analysis of multimodal omics studies and reveals more powerful biological insights from limited cohort sizes.

## Abstract (web summary)

COMET, an artificial intelligence method that improves the analysis of small medical studies using large clinical databases, has been created. COMET can help develop better artificial intelligence tools and identify key biomarkers across many diseases, potentially changing medical research.

## Main

Rapid advancements in omics technologies have revolutionized biological understanding. Transcriptomic, metabolomic, proteomic and other biomolecular assays now enable the cost-effective measurement of vast numbers of analytes from a single sample. Although these assays produce high-dimensional data, clinical and budgetary constraints limit the size of most omics study cohorts, resulting in poor replication of findings^1–4^. Hence, there is a need for innovative analytical approaches to improve the analysis of high-dimensional data from these small cohort studies.

Although statistical tools like the Benjamini–Hochberg procedure address false-positive rates in univariate analyses, fewer methods exist for machine learning, where false positives manifest as overfit models^5^. Some recent approaches utilize existing knowledge about which features are expected to be important, which is used as a prior on the feature’s weight in the machine learning model^6,7^. Other approaches use transfer learning, a technique in which a machine learning model is learned from a pretraining dataset that is subsequently used to analyse a smaller dataset of interest^8,9^. More modern deep learning approaches have also been applied to traditional statistical frameworks, like the Cox proportional hazards model, which can be used to analyse time-to-event data, especially in datasets with censored patients^10^. Although these methods have enhanced our ability to analyse high-dimensional omics data, they primarily focus on learning from omics data alone or informative metadata. In this work, we utilize electronic health record (EHR) data to improve omics data analysis. EHR data are becoming increasingly accessible through both public datasets (like MIMIC^11^ and UK Biobank^12^) and proprietary medical centre databases (enabled by standards like Meaningful Use Stage 2 and Observational Medical Outcomes Partnership (OMOP)^13–15^).

Existing multimodal machine learning methods combine data modalities through early fusion (at the feature level), intermediate fusion (combining latent representations after modality-specific data processing) or late fusion (combining modality-specific predictions)^16^. There are also generalized frameworks that can blend these approaches^17^. However, it is generally easier to access EHR data for large populations, which creates challenges in EHR–omics integration: early and intermediate fusion generally require complete data across modalities, potentially excluding many patients, whereas late fusion approaches struggle to learn cross-modal interactions^18,19^.

We introduce clinical and omics multimodal analysis enhanced with transfer learning (COMET), a deep learning architecture and transfer learning protocol that utilizes transfer learning from large, observational EHR databases to improve the analysis of multimodal datasets from omics studies. By leveraging pretraining, COMET uses all available EHR data to learn a powerful machine learning model; by transferring these weights into a multimodal architecture, COMET can learn interactions across modalities. We show that COMET achieves state-of-the-art predictive modelling results and enables more robust biological discovery in two different clinically relevant tasks. We demonstrate that the EHR pretraining component of COMET has a regularization effect across the entire network, improving the model’s performance and its ability to learn generalizable biology. Our work has broad implications for changing the way that we analyse data from omics studies by utilizing existing EHR data and can improve our ability to make discoveries without changes to study design or increasing cohort size.

## Results

**Fig. 1:**
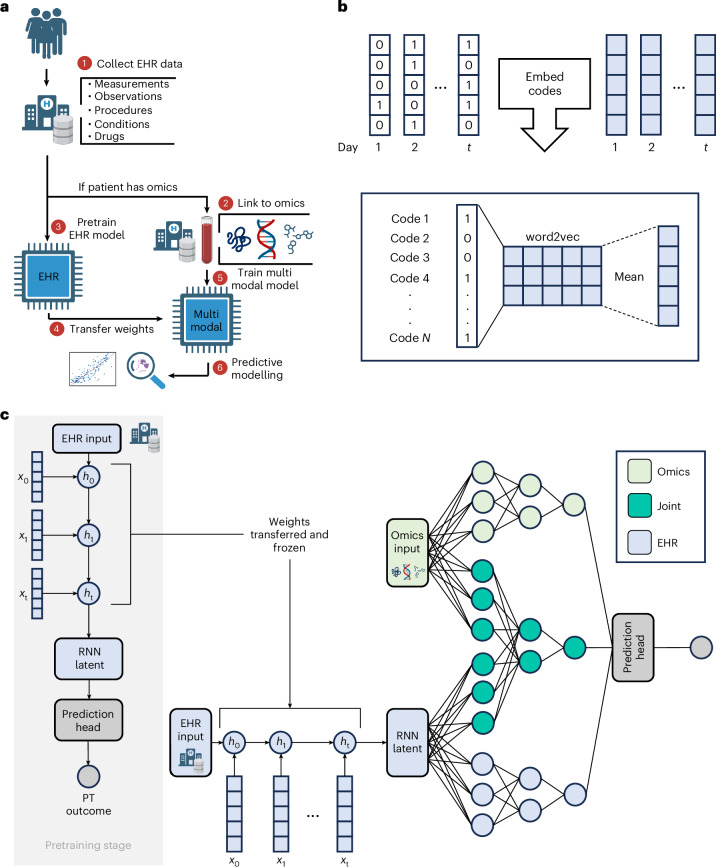
COMET is a deep learning framework that uses large, observational EHR databases and transfer learning to improve the analysis of small datasets from omics studies. **a**, The input to COMET is EHR data and (for a subset of patients) paired, tabular omics data. The patients who only have EHR data are used to pretrain (PT) a neural network predict patient outcomes using only EHR data. The weights from this EHR network are transferred to a multimodal neural network used to analyse both EHR and omics data; the neural network is used for predictive modelling and post hoc analysis of the network is used for biological discovery. The COMET framework is flexible and can be used to predict any continuous or binary outcome. **b**, One-hot encoded vectors of EHR data (shown in white) are converted into embeddings (shown in blue) using word2vec; the embeddings for each code that occur within a particular day are averaged to compute sequential, summary embeddings. **c**, COMET uses a multimodal deep learning architecture to analyse both EHR data and omics data. Only EHR data are used in the pretraining stage; the core architecture is an RNN with gated recurrent units. After pretraining, the RNN weights are frozen and transferred into a multimodal architecture that analyses both EHR and omics data. Panel **a** created with BioRender.com.

In general, COMET can be applied when EHR data are available for a large cohort of patients, and omics data are available for a smaller sub-cohort. A model trained on patients with only EHR data (the ‘pretraining cohort’) has its weights transferred to a multimodal network, which is further trained and tested on the smaller population with both EHR and omics data (the ‘omics cohort’) (Fig. 1a). COMET consists of three parts: a method to embed longitudinal EHR data^20^ (Fig. 1b), pretraining and multimodal modelling (Fig. 1c). Here we used COMET to analyse two independent cohorts, one pregnancy cohort from Stanford Health Care and one cancer cohort from the UK Biobank. In each cohort, we demonstrate COMET’s state-of-the-art performance for a clinically meaningful predictive modelling task: days to the onset of labour or three-year all-cause mortality, respectively. We perform all the modelling experiments 25 times with different train, test and validation splits, and compute the performance metrics using the average predictions from the validation set.

### COMET accurately predicted days to the onset of labour

**Fig. 2:**
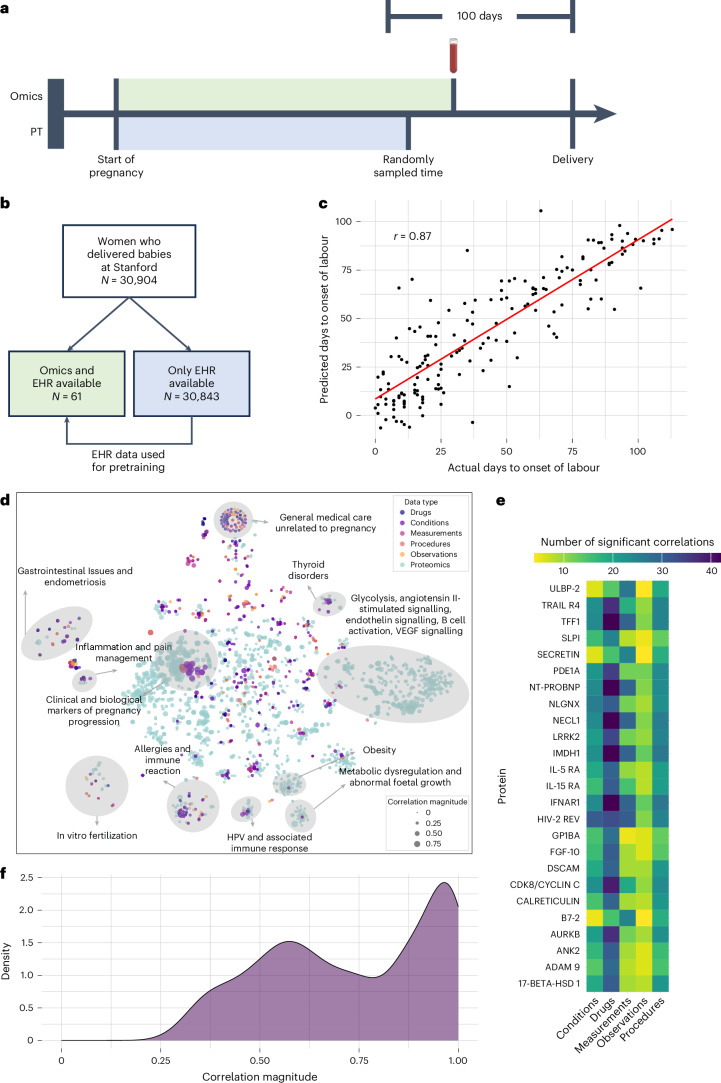
Multimodal data reveals EHR–proteomics interactions related to pregnancy progression and time to the onset of labour. **a**, For patients with proteomics data, input features were constructed using EHR data from the beginning of pregnancy up to the proteomics sampling time (shaded in green); for patients in the pretraining (PT) cohort without proteomics data (and therefore without a sampling time), we randomly sample a time point at which to cut off the EHR data (features are constructed from the time shown in blue; we use days from that time point until labour as our predictive modelling task). **b**, We utilized data from women who gave birth at Stanford, and split the women into two populations based on whether or not they had omics data available. **c**, Predictions using the COMET framework are compared with actual days to the onset of labour, with the regression line shown in red. **d**, *t*-SNE visualization of the onset of labour data. The dots represent individual features and are coloured based on modality; dots are sized based on the feature’s univariate Pearson correlation with days to the onset of labour. The clusters with only protein variables are annotated based on gene ontology enrichment analysis and clusters containing both clinical and protein variables are annotated based on clinical themes. **e**, Heat map showing the number of significant correlations (after Bonferroni correction) between the EHR features and proteins; the 25 proteins with the greatest number of statistically significant correlations with EHR features are shown. **f**, Distribution of the maximum absolute correlation of each individual EHR feature with all proteins in the onset of labour data.

We first applied COMET to predict days to the onset of labour in a population of pregnant people (*n* = 30,904 patients) who delivered newborns at Stanford from 2013 to 2021. The EHR data for all individuals were extracted from the Stanford STARR OMOP database. For a subset of pregnant patients (*n* = 61 patients, the omics cohort), multiple blood (plasma) samples were collected throughout the last 100 days of their pregnancy and used to generate a targeted proteomics dataset that measured 1,317 different proteins^21^. We used the EHR data from the beginning of pregnancy up to the time of the blood sampling and aimed to predict days to the onset of labour (from the time of sampling). For the patients with only EHR data (*n* = 30,843 patients, the pretraining cohort), there is no sampling time (as these patients do not have proteomics data). Therefore, we randomly chose a time point within the last 100 days of pregnancy, used the EHR data up to the sampled time as features and predicted days to the onset of labour from the sampled time as the pretraining task (Fig. 2a,b).

We embedded longitudinal EHR data using word2vec, averaging embeddings for codes within each day. After pretraining the EHR-only architecture with the data from the pretraining cohort, we transferred weights to the full multimodal architecture, which was trained to make predictions on the omics cohort. The Pearson correlation between the predicted days to the onset of labour and actual days to the onset of labour using COMET was strong, indicating that COMET can make highly accurate predictions in small cohorts with high-dimensional data (*r* = 0.868, 95% confidence interval (CI) [0.825, 0.900], *P* = 3.9 × 10^–53^, root mean square error (r.m.s.e.) = 16.0) (Fig. 2c). Agreement is measured using Lin’s concordance correlation coefficient and reported in Supplementary Table 1, which confirms that COMET’s predictions align well with actual time-to-onset values without systematic bias.

We compared COMET with baseline models using only EHR data, only proteomics data or both (‘joint baseline’). These baselines use only omics cohort data without pretraining, with architectures matching the corresponding parts of COMET. The EHR-only baseline uses only the EHR part of the network (Fig. 1c, light blue). The proteomics-only baseline uses only the omics part of the network (Fig. 1c, light green). Last, the joint baseline uses both data modalities and matches the full COMET architecture. The only difference between the joint baseline and the COMET framework is that the joint baseline excludes the pretraining stage. The EHR-only baseline performed the worst (*r* = 0.768, 95% CI [0.699, 0.823], *P* *=* 1.55 × 10^–34^, r.m.s.e. = 20.4 days), and was slightly outperformed by the proteomics-only baseline (*r* = 0.796, 95% CI [0.733, 0.845], *P* = 1.3 × 10^–38^, r.m.s.e. = 20.2 days). The joint baseline was the highest-performing baseline (*r* = 0.815, 95% CI [0.757, 0.860], *P* = 7.8 × 10^–42^, r.m.s.e. = 18.4 days), but is still inferior to COMET. To confirm that COMET provides benefit across different omics modalities, we ran a similar set of experiments using metabolomics for the same cohort and show that predictive modelling results with COMET (*r* = 0.839, 95% CI: [0.782, 0.881]) exceed the performance of predictions from metabolites alone (*r* = 0.758, 95% CI: [0.678, 0.820]). Supplementary Table 2 lists the full results.

We have also compared COMET with baselines using ridge regression, and computed performance for EHR-only, proteomics-only and joint baselines. To determine if we could incorporate EHR pretraining in different ways, we trained an EHR-only ridge regression model using data from the pretraining cohort and use an adapted version of ridge regression inspired by another work^7^ that incorporates the coefficients from the pretrained model as priors on the weight in the joint (that is, multimodal) model. Incorporating pretraining improves the Pearson correlation of the joint baseline (from *r* = 0.572, 95% CI: [0.461, 0.665] to *r* = 0.799, 95% CI: [0.737, 0.847]), with COMET still outperforming all approaches (full results are in Supplementary Table 3).

Last, we wanted to compare between COMET’s word2vec and a recurrent neural network (RNN)-based approach to compute a latent representation of EHR data to an approach that utilizes a transformer, including learning token embeddings in an end-to-end manner (which we call COMET Transformer). There is strong correlation between the predictions from COMET and COMET Transformer (*r* = 0.94). The Pearson correlation for the transformer variant is 0.848 (95% CI: [0.800, 0.885]), slightly underperforming COMET (full results are listed in Supplementary Table 4). Taken together, these results demonstrate the value of incorporating pretraining regardless of the model architecture, and the superior ability of COMET to predict days to the onset of labour.

### Analysis of COMET EHR–proteomics feature correlations revealed biological insights into pregnancy

COMET’s superior performance prompted us to further investigate the relationships between EHR and proteomics features, with the goal of gaining deeper insights into the complex biological processes during pregnancy. First, we used *t*-distributed stochastic neighbour embedding (*t*-SNE) to visualize the multimodal data by projecting the correlation matrix into two dimensions; features close together in this space have similar correlations with all other variables (Fig. 2d). We annotated these clusters based on the medical concepts that the EHR and/or protein features within each cluster represent. For example, the ‘metabolic dysregulation and abnormal foetal growth’ cluster contains clinical codes representing abnormal glucose tolerance in the mother, maternal obesity and excessive foetal growth. It also contains proteins like betacellulin and oncostatin M, which are known to play a role in glucose homeostasis and insulin sensitivity^22,23^.

We similarly visualized each EHR modality individually, and used lines to connect significantly correlated cross-modality variables (Supplementary Fig. 1). These visualizations revealed that there are many EHR variables that are highly correlated with other features (including proteins), suggesting redundancy in information across modalities. However, 46.5% of proteins have no significant correlations with any EHR features, indicating that the proteomics data also provide some complementary information (Supplementary Fig. 2).

Several proteins showed high numbers of significant correlations with EHR variables (Fig. 2e), such as interferon alpha and beta receptor subunit 1, which correlates with multiple infection-related variables, aligning with its known role in immune function. To investigate the additional value of the clinical data, we performed a complementary analysis and computed the correlation of each clinical variable with all proteins and plotted the distribution of maximum correlation (Fig. 2f). These analyses show both overlapping and unique information across both modalities. The pretraining stage of COMET allows the RNN to extract the most useful information and avoids the inclusion of redundant, highly correlated EHR features, which may contribute to its superior performance compared with the baseline models.

### COMET aligned EHR and proteomics data

**Fig. 3:**
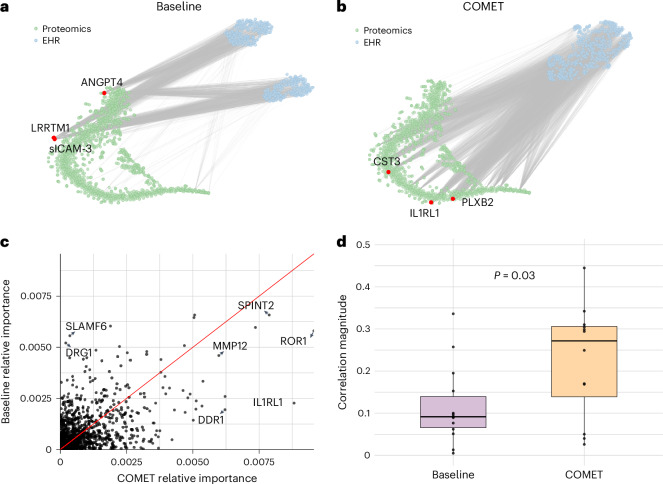
COMET induced alignment between EHR latent representations and proteomics data. **a**, *t*-SNE visualization of the proteomics data and EHR latent representation in the joint baseline models; lines connect statistically significantly correlated proteins and dimensions of the EHR latent representation. The red dots represent three proteins with the greatest number of statistically significant correlations with the dimensions of the EHR latent representation. sICAM-3, soluble intercellular adhesion molecule 1; LRRTM1, leucine-rich repeat transmembrane neuronal protein 1; ANGPT4, angiopoietin-4; CST3, cystatin C; PLXB2, plexin-B2; IL1RL1, interleukin-1 receptor-like 1. **b**, *t*-SNE visualization of the proteomics data and EHR latent representation in the COMET models. The lines connect statistically significantly correlated proteins and dimensions of the EHR latent representation. The red dots represent three proteins with the greatest number of statistically significant correlations with the dimensions of the EHR latent representation. **c**, Comparison of protein feature importance in the COMET models and joint baseline models. **d**, Distribution of absolute correlations between protein abundance and days to the onset of labour in an external dataset (*n* = 12 correlations from important proteins in the baseline model and *n* = 14 correlations from important proteins in the COMET models). The box plots show the median (centre line), 25th and 75th percentiles (box bounds), with whiskers extending to the most extreme data points within 1.5 times the interquartile range from the box edges. The difference between the average absolute correlations is 0.109 (95% CI: [0.0134, 0.2052]) with a two-sided *t*-statistic of –2.39 (*P* = 0.0276) with an estimated degree of freedom of 19.1.

We examined EHR–proteomics relationships through the EHR latent representation, visualizing correlations between each of the 400 latent dimensions and each protein (Fig. 3a,b). There were 3,201 significant correlations (after multiple hypothesis test correction) between the dimensions of the EHR latent representations learned in the joint baseline models and all proteins. COMET’s EHR latent representations showed 5,364 significant correlations, indicating better alignment of the EHR and proteomics data. The increased alignment suggests that the information COMET learns from the EHR data more closely captures the underlying biological processes of the patient.

This pattern was the strongest in proteins most correlated with EHR latent representation dimensions. Using COMET, interleukin-1 receptor-like 1 (also known as the suppression of tumourigenicity 2 protein), cystatin C and plexin-B2 showed significant correlations with 76%, 68% and 68% of the dimensions, respectively. These proteins are known to play a role in pregnancy progression and labour timing, and are consistent with discoveries from previous studies^24–28^. The EHR latent representation’s high correlation with these proteins suggests that it was capturing meaningful information about the patients’ underlying biological state, potentially contributing to improved predictive modelling performance. By contrast, the joint baseline models do not exhibit this phenomenon. In the baseline experiments, the proteins most correlated with the EHR latent representation were soluble intercellular adhesion molecule 1, leucine-rich repeat transmembrane neuronal protein 1 and angiopoietin-4. Although angiopoietin-4 does have a known association with pregnancy progression, the other two proteins are primarily known for other biological functions unrelated to pregnancy, suggesting that the EHR latent representation from the baseline models does not reflect the underlying pregnancy biology as strongly. Further discussion of these proteins is provided in Supplementary Note 1.

### COMET identified proteins associated with labour onset timing

Last, we computed the feature importance for each protein using integrated gradients to understand how the alignment between the EHR latent representation and the proteins influenced the features ultimately used by the models to make their predictions (Fig. 3c; full feature importance is provided in Supplementary Data). The proteins with greater feature importance in the COMET models are known to be associated with gestational age, foetal development or pregnancy complications, all of which have implications for time to labour. Conversely, the proteins that are more important in the joint baseline models have no known role in pregnancy. We expand on the known biological role of these proteins in Supplementary Note 2 and further validate the relevance of the important proteins in the COMET models by computing the correlation between these proteins and days to the onset of labour in an external dataset (Fig. 3d). The average Pearson correlation magnitude for the proteins more important in COMET was 0.22 (s.d. = 0.13), whereas the average for the proteins less important with COMET was 0.12 (s.d. = 0.09). These analyses suggest that COMET improves predictive modelling not only through learning a more biologically meaningful representation of the EHR data but also helps the model learn accurate biology.

### COMET improved cancer prognosis prediction

**Fig. 4:**
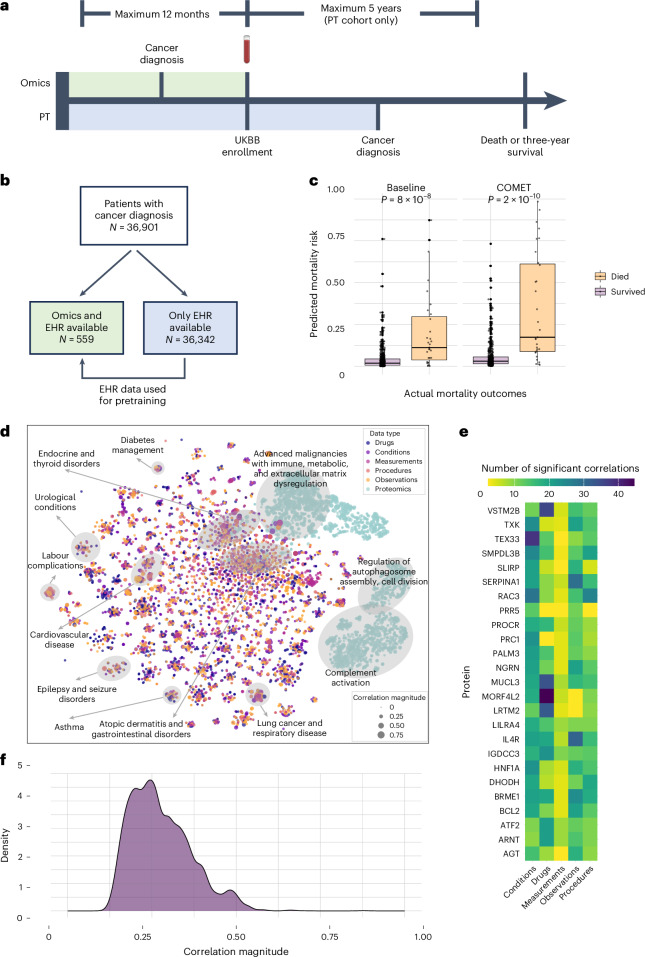
Multimodal data provided insights into cancer mortality risk. **a**, For patients with proteomics data, we construct input features from all EHR data up to the sampling time (shaded in green); for patients without proteomics data, we use EHR data up until cancer diagnosis (shaded in blue). **b**, We utilized data from patients with a cancer diagnosis in the UK Biobank (UKBB), and split the population into two groups based on whether or not they had omics data available. **c**, Predictions from COMET were better than predictions from the highest-performing baseline (*n* = 559 predictions). The box plots show the median (centre line), 25th and 75th percentiles (box bounds), with whiskers extending to the most extreme data points within 1.5 times the interquartile range from the box edges. The difference in mean for the baseline predictions was 0.089 (95% CI: [0.0469, 0.1277]) with a two-sided Wilcoxon rank sum statistic of 3,431 (*P* = 8.37 × 10^–8^). The difference in mean for the COMET predictions was 0.149 (95% CI: [0.0892, 0.3084]) with a two-sided Wilcoxon rank sum statistic of 2,537 (*P* = 1.54 × 10^–10^). **d**, *t*-SNE visualization of cancer mortality data. The dots represent individual features and are coloured based on modality. They are sized based on univariate correlation with cancer mortality. The clusters with only protein variables are annotated based on GO enrichment analysis and clusters containing both clinical and protein variables are annotated based on clinical themes. **e**, Heat map showing the number of significant correlations (after Bonferroni correction) between the EHR features and all proteins. **f**, Distribution of the maximum absolute correlation between each EHR feature and all proteins in the cancer mortality data.

To show the generalizability of our COMET framework, we next applied it to a different prediction problem in an independent population. We used COMET to predict the three-year cancer mortality from a population of cancer patients in the UK Biobank (*n* = 36,901 patients)^11^. The studied population consisted of all the patients who received a diagnosis of any type of cancer (determined by the presence of an ICD10 code beginning with C) within 5 years of enrolment in UK Biobank, or up to 12 months prior. A subset of these patients had blood samples collected when they enrolled in the UK Biobank study, which were analysed for the proteomics data^29^. We included these patients in our omics cohort if they had their samples collected within 12 months following their initial cancer diagnosis (*n* = 559 patients, the omics cohort). For patients with proteomic data, we used EHR data from the time of sampling and earlier as features; for other patients (*n* = 36,342 patients, the pretraining cohort), we used EHR data from the time of cancer diagnosis and earlier (Fig. 4a,b).

When using COMET to predict three-year cancer mortality by using the pretraining cohort to pretrain the EHR part of the model and transferring those weights to a multimodal model to make predictions on the omics cohort, it demonstrates superior performance compared with all the baselines (area under the receiver operating characteristic curve (AUROC) = 0.842, 95% CI: [0.744, 0.922], *P* = 0, area under the precision–recall curve (AUPRC) = 0.504, 95% CI: [0.341, 0.670], *P* = 0; Fig. 4c). The prevalence of three-year mortality is 5.5% in the omics cohort. The baselines have the same design as the onset of labour analyses (see the ‘COMET accurately predicted days to the onset of labour’ section for details). The joint baseline performed the best (AUROC = 0.786, 95% CI: [0.664, 0.882], *P* = 0, AUPRC = 0.365, 95% CI: [0.217, 0.555], *P* = 0). The EHR-only (AUROC = 0.749, 95% CI: [0.636, 0.843], *P* = 0, AUPRC = 0.205, 95% CI: [0.122, 0.349], *P* = 0) and proteomics-only (AUROC = 0.737, 95% CI: [0.634, 0.838], *P* = 0, AUPRC = 0.325, 95% CI: [0.179, 0.495], *P* = 0) baselines also show some signal for predictive modelling. Agreement is measured using Cohen’s kappa and is reported in Supplementary Table 5, demonstrating consistent and reliable classification performance that exceeds all baselines.

Like the onset of labour experiments, we compared the performance of COMET with a logistic regression baseline, including an adaptation that incorporates prior knowledge that similarly shows a benefit from the baseline AUPRC of 0.263–0.279 when incorporating priors from the pretrained model. Full results are listed in Supplementary Table 6, which show that COMET exceeds all logistic regression baselines, including the adaptation that incorporates priors from pretraining. We also ran the experiments using COMET Transformer, which again show a strong correlation between predictions (*r* = 0.72) with COMET outperforming COMET Transformer (Supplementary Table 7). Regardless of the model architecture, predictive modelling performance improved when pretraining was included, and the performance of COMET exceeds all other approaches.

### Multimodal data uncovered biology of cancer prognosis

We used *t*-SNE to visualize the correlation matrix among all pairs of variables across modalities to better understand their relationships (Fig. 4d). In contrast to the onset of labour data, there was less overlap between the proteomics data and the EHR data modalities. However, we do see significant correlations between the proteomics data and EHR data modalities when visualizing a correlation network with each modality individually projected into two dimensions (Supplementary Fig. 3).

To gain insights into this phenomenon, we computed the number of significant correlations each protein variable has with all the EHR variables (Fig. 4e). Among all the proteins, mortality factor 4-like protein 2 had the greatest number of correlations with EHR variables, especially drug prescriptions. Mortality factor 4-like protein 2 has been associated with tumour dynamics and treatment response, which may explain its high correlation to drug orders^30^. We found a large proportion of the proteins in cancer patients (65.9%) had no significant correlation with any of their EHR variables (Supplementary Fig. 4). We computed the correlation of each EHR feature with all proteins and computed the maximum correlation across all proteins for each EHR feature (Fig. 4f) and found many EHR features with low correlations to all proteins in the cancer patients. This finding reiterates the value of including multiple data modalities in our analysis. When looking at the strong correlations between EHR features and proteins, it allowed us to uncover interesting relationships across data modalities. For example, a diagnosis of chronic B cell lymphocytic leukaemia has the highest correlation with lymphocyte-activation gene 3 protein intensity (*r* = 0.46, 95% CI: [0.333, 0.571], *P* = 8.4 × 10^–^^31^); lymphocyte-activation gene 3 is an immune checkpoint that is expressed on leukaemia cells and has been shown to be an effective prognostic marker (Supplementary Fig. 5)^31^.

### COMET EHR representations reflected known cancer biology

**Fig. 5:**
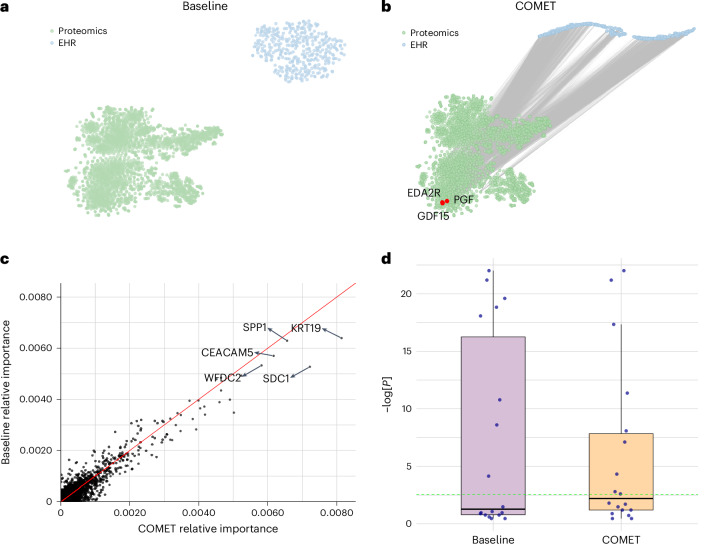
COMET induced alignment between EHR latent representations and proteomics data, and produced models that are more biologically aligned with known pregnancy biology. **a**, *t*-SNE visualization of the proteomics data and EHR latent representation in the joint baseline models. The lines connect statistically significantly correlated proteins and dimensions of the EHR latent representation. The red dots represent three proteins with the greatest number of statistically significant correlations with the dimensions of the EHR latent representation. **b**, *t*-SNE visualization of the proteomics data and EHR latent representation in the COMET models. The lines connect statistically significantly correlated proteins and dimensions of the EHR latent representation. **c**, Comparison of protein feature importance in the COMET models and joint baseline models. **d**, Distribution of univariate *P* values (from a *t*-test) comparing protein levels based on three-year mortality in an external dataset (*n* = 18 *P* values from important proteins in the baseline model and *n* = 18 *P* values from important proteins in the COMET models). The green dotted line represents the Bonferroni-adjusted significance threshold.

We again visualize the relationship between EHR latent representation and proteomics data (Fig. 5a,b). The dimensions of the EHR latent representation learned in the joint baseline experiments have no significant correlations with any proteins, whereas the dimensions of the EHR latent representation from COMET had 7,591 statistically significant correlations, showing that this alignment effect occurs across datasets. All the proteins with the greatest number of significant correlations with the COMET EHR latent representation have been shown to be prognostic biomarkers for cancer. We elaborate on these proteins in Supplementary Note 3. These findings demonstrate that COMET not only effectively aligns the EHR and protein data but also reveals biologically meaningful correlations that are consistent with known cancer prognostic markers, underscoring the potential of this approach for identifying clinically relevant biomarkers and therapeutic targets across diverse datasets.

### COMET models validated established cancer prognostic markers

Proteins with higher feature importance in COMET models aligned with known prognostic biomarkers (Fig. 5c, full feature importance is provided in Supplementary Data). We elaborate on these proteins in Supplementary Note 4. We further validated that the proteins more important in the COMET models are more highly associated with mortality than the proteins that are more important in the baseline models (Fig. 5d). We found that 9 out of the 18 matching proteins that were most important in the COMET models are statistically significantly associated with mortality status, whereas only 8 were from the joint baseline models. Furthermore, the median *P* value for the COMET proteins was lower. These findings further validate that COMET models better align with known biology.

### COMET acted as a form of regularization by initialization

**Fig. 6:**
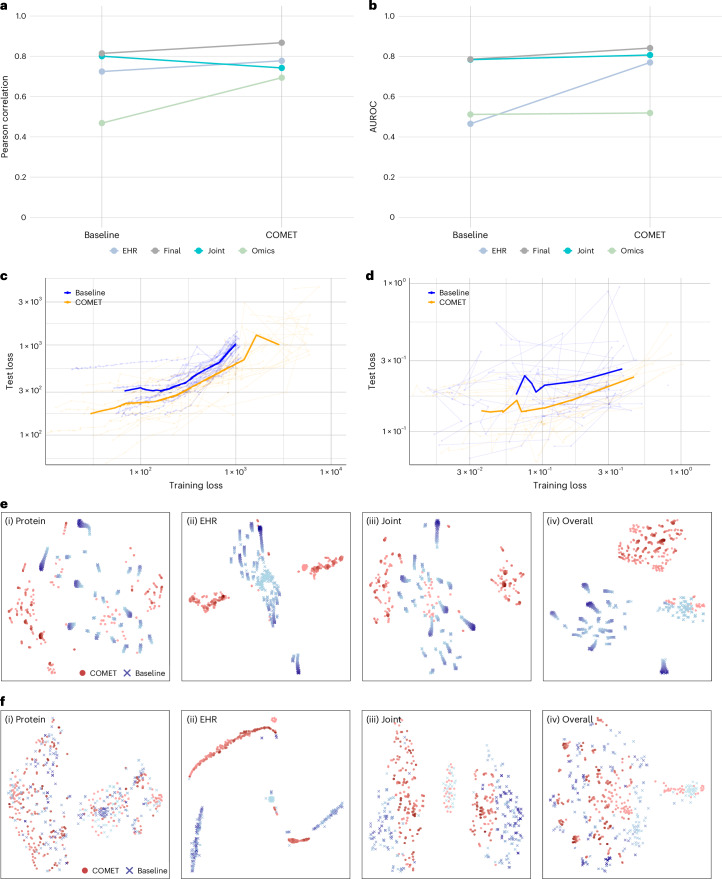
COMET acted as a form of regularization, allowing the neural network to access parts of the parameter space that would not be accessible otherwise. **a**, Pearson correlation of the values at each intermediate node with days to the onset of labour in the joint baseline model compared with COMET. **b**, AUROC of the values at each intermediate node for predicting three-year mortality in the joint baseline model compared with COMET. **c**, Training loss versus test loss for each iteration of the onset of labour experiment at each epoch, comparing the joint baseline with COMET; the mean loss is shown in bold. **d**, Training loss versus test loss for each iteration of the cancer mortality experiment at each epoch, comparing the joint baseline with COMET; the mean loss is shown in bold. **e**, Visualization of the parameter space for joint baseline and COMET models to predict days to the onset of labour; each point represents the parameters at one epoch during training. Earlier epochs are shown in lighter colours. Protein parameter space (i), EHR parameter space (ii), joint parameter space (iii) and overall parameter space (iv). **f**, Visualization of the parameters for joint baseline and COMET models to predict cancer mortality. Each point represents the parameter space at one epoch during training. Earlier epochs are shown in lighter colours. Protein parameter space (i), EHR parameter space (ii), joint parameter space (iii) and overall parameter space (iv).

To better understand which part of the network was responsible for the predictive modelling improvements, we looked at the performance of the intermediate nodes in the penultimate layer of the network (Fig. 6a,b). As expected, we saw improvements in the EHR node with COMET, presumably due to the additional EHR data used to pretrain the model. The improvements in the biological representations discussed above also suggest that the proteomics and/or joint nodes may also have improvements. Indeed, we see that effect (from the proteomics node in the onset of labour analysis and from the joint node in the cancer analysis). These findings support the hypothesis that COMET not only improves the model’s ability to learn from the EHR data but also from the the omics data. We also show that the weights in the omics and joint parts of the network are a function of these transferred weights (Supplementary Note 5); therefore, such a finding is also supported theoretically.

To understand the mechanism of this improvement, we compared the training loss against the test loss between the COMET models and the baseline models (Fig. 6c,d). We observed that the test loss was lower for any given training loss when using COMET, suggesting that COMET improves generalizability, potentially by acting as a form of regularization. We explored how this regularization effect impacted the actual model parameters.

By visualizing the parameter space (Methods), we can see that the COMET models occupy separate parts of the parameter space compared with the baseline models (Fig. 6e,f). This suggests that the regularization effect allows parameters to converge to a part of the parameter space that leads to more generalizable and more biologically accurate models. The paths of each of the 25 iterations of the models through the parameter space throughout training are visualized in Supplementary Figs. 6–13. In conclusion, the improved performance of the COMET models occurs due to the model’s ability to better learn from both EHR and omics data, enabled by the regularization effect that is a result of COMET’s initialization of weights in the RNN from transfer learning.

## Discussion

We demonstrated COMET’s ability to improve predictive modelling across various tasks through pretraining and transfer learning, which enable access to previously unreachable parts of the parameter space. COMET results in better regularized models that more accurately reflect known biology and encourage EHR–omics alignment. Importantly, we show that using EHR pretraining improves the predictive modelling performance across different deep learning architectures, as well as ridge regression and logistic regression baselines. By integrating EHR with omics data, COMET pioneers multimodal analysis in biomedicine, advancing beyond traditional single-modality approaches^19,32–35^. To our knowledge, COMET is the first approach to utilize EHR transfer learning to improve the analysis of omics data.

COMET improves biological modelling through the influence of the RNN’s pretrained weights on gradient computation in the joint and omics network components through backpropagation. Consequently, COMET models identified biologically relevant proteins for specific health outcomes. In the onset of labour models, it highlighted proteins crucial for immune regulation, placental development and pregnancy complications (interleukin-1 receptor-like 1, cystatin C, SPINT2, DDR1, VEGFR sR3 and MMP12). For cancer mortality, it identified proteins involved in tumour proliferation and microenvironment modulation (CEACAM5, KRT19 and SDC1), demonstrating COMET’s capacity to uncover meaningful molecular mechanisms.

There are several limitations to this study. Although we have shown that COMET can be applied to both regression and classification tasks, further research is needed to determine if it can generalize to different architectures. It is unknown if COMET will lead to similar improvements if the omics architecture is more complex, as necessary for some omics modalities (for example, spatial transcriptomics). Additionally, the EHR data are OMOP extracts, which are manually mapped and may contain errors. Future work will focus on assessing generalizability to other data structures and architectures. Last, the COMET framework requires labels in the EHR pretraining dataset—future work will explore self-supervised pretraining tasks, particularly given recent work showing that EHR foundation models can directly predict protein expression levels^36^.

COMET advances multimodal biomedical data integration by leveraging EHR data to enhance omics analysis, improving both predictive modelling and biological discovery. Moving beyond simple case-control categorizations, it captures nuanced disease states that may be obscured by reductionist study designs. Its regularization properties improve model generalizability and robustness, enhancing potential clinical translation. As multimodal biomedical data availability grows, COMET provides a foundation for unravelling complex relationships between clinical phenotypes and molecular mechanisms and can change how we analyse data from omics studies.

## Methods

### Datasets

The two cohorts utilized in the study are from real-world clinical studies. The first dataset comprises serial blood samples collected from 61 women at Stanford throughout the last 100 days of pregnancy^20^. All pregnancies had spontaneous onset of labour (that is, C-section and medical induction of labour cases were excluded). Demographics of the patients are described in Supplementary Table 8. Each patient had two or three samples collected. There are a total of 171 samples used in the analyses. Train/test/validation splits happen at the patient level; therefore, all the samples from a patient are contained in the same data split. The plasma samples were analysed to measure 1,317 proteins using an aptamer-based platform. We excluded 12 proteins that were used as controls; thus, the analyses only considered 1,305 proteins. Linking data from the study to EHR records was approved by the institutional review board (Stanford IRB #39225).

The UK Biobank is a large-scale biobank containing in-depth biomedical and health information from over half a million UK participants^37^. A subset of participants provided blood samples that were analysed using an aptamer-based platform to measure a median of 2,894 proteins from 53,058 participants^28^. Demographics of the participants are described in Supplementary Table 9. We only use the proteomics data generated from the blood sample collected at the time of UK Biobank study enrolment; hence, each patient in our analysis has one blood sample.

### Extraction of EHR data and pretraining cohort

EHR data for both datasets are provided in the OMOP format^14^. For patients in our dataset, we extract all data from the measurement, observation, drug_exposure, condition_occurrence and procedure_occurrence tables. We remove rows that have a concept_id of 0.

For the omics cohort in the onset of labour dataset, we derive data from the beginning of pregnancy (defined as 280 days before childbirth) up until the time of omics sampling (Fig. 2a). For the pretraining cohort in the onset of labour dataset, we simulate the design of the original omics study in which we sample a random date between childbirth and up to 100 days prior. We then use EHR data from that date and earlier, up to the beginning of pregnancy.

For the omics cohort in the cancer mortality dataset, we extract EHR data from the time of proteomics sampling and earlier (Fig. 4a). For the pretraining cohort in the cancer mortality dataset, we compute a time of cancer diagnosis based on the first occurrence of any ICD10 code that begins with C. We extract data from that time of cancer diagnosis and earlier. We use the death table in OMOP to identify mortality.

### Embedding process

#### Onset of labour

To make the EHR data more amenable to analysis in a deep learning model, we first learn embeddings for each of the codes. To do this, we extract unique concept codes across the EHR data tables mentioned above. We then group the codes by patient and by day. All codes that occur for a patient within a given day are considered ‘words’ in a ‘sentence’. They are randomly shuffled as the specific time stamps of many EHR variables (especially conditions) are not reliable. We then use word2vec to learn 400-dimensional embeddings for each ‘word’ (concept codes) of these ‘sentences’ (sequence of codes representing all the clinical events within a particular day)^38^. A separate word2vec model is learned for the pretraining and omics cohorts. After the embeddings are learned, we take the mean of the embeddings for codes that occurred within a specific day. We now have one ‘summary embedding’ for all the EHR data within a particular day for each patient. These sequences of summary embeddings are what will be fed sequentially into the RNNs for predictive modelling. We use a maximum of 32 days of data, starting from the most recent dates.

#### Cancer mortality

We use the same process as that described above.

### COMET deep learning architecture

The architecture for the experiments slightly differs as one is a regression task and the other is a classification task. Generally, both architectures have an ‘EHR’ component, a ‘joint’ component and an ‘omics’ component.

#### Onset of labour

The EHR component of the network consists of an RNN with gated recurrent units, followed by a single linear layer that takes the output of the last RNN layer and generates a single, EHR-based prediction. The number of layers, hidden dimension and dropout are chosen as hyperparameters. This architecture is used for the EHR-only baseline. The omics component of the network is a single linear layer that takes the omics as input and generates a single, omics-based prediction. This part of the architecture is used for the omics-only baseline. The joint layer is a single linear layer that takes the concatenated EHR latent representation and omics data and generates a single, joint prediction. The final layer of the network is a single linear layer with no bias that combines the three predictions into a final prediction. The network is trained by minimizing the mean squared error loss:

$${{\rm{MSE}}}=\frac{1}{N}\mathop{\Sigma }\limits_{i=1}^{N}{(\;{y}_{{{\rm{true}}}}-{y}_{{{\rm{pred}}}})}^{2}$$

#### Cancer mortality

The architecture for the cancer mortality dataset is similar to the above with minor changes as the model is used for classification instead of regression. We use a multilayer perceptron with one hidden layer and rectified linear unit activation functions between the input and hidden layers, and include a sigmoid at the end of the network and at the end of the omics-only part of the model. We slightly vary the architecture to demonstrate that COMET is not architecture-specific and the framework can be beneficial across multiple deep learning architecture designs. The network is trained by minimizing the binary cross-entropy loss.

$${{\rm{BCE}}}=-\frac{1}{N}\mathop{\Sigma }\limits_{i=1}^{N}\left(\;{y}_{{{\rm{true}}}}\log \left[{y}_{{{\rm{pred}}}}\right]+\left(1-{y}_{{{\rm{true}}}}\right)\log \left[1-{y}_{{{\rm{pred}}}}\right]\right).$$

### COMET hyperparameter details

To determine the hyperparameters, we use a threefold cross-validation and grid search. Within the two training folds, we take 20% of the data as a test set for early stopping. We assess the performance of each hyperparameter set via grid search on the validation set and choose the hyperparameter set that gives the lowest average loss on the validation sets across the threefold cross-validation. These hyperparameters are used for all the subsequent experiments, including those with different train, test and validation splits. For the EHR part of the network, the parameter grid is as follows: learning_rate, {1 × 10^–1^, 1 × 10^–^^2^, 1 × 10^–3^, 1 × 10^–4^}; dropout, {0.1, 0.2, 0.3, 0.4, 0.5}; lr_decay, {1 × 10^–1^, 1 × 10^–2^, 1 × 10^–3^, 1 × 10^–4^}; layers, {2, 4}; hidden_dim, {400}. The batch size is fixed at 512 for the pretraining cohort and 16 for the omics cohort. For the proteomics-only experiment, we separately optimize the learning rate and lr_decay from the same range. From the joint experiments, we optimize learning rate, dropout and lr_decay, but fix the number of layers and hidden dimension as the optimal weights chosen from the EHR-only experiments. When we transfer the weights, we fix the number of layers and dropout as the optimal values from the pretraining experiments. In the cancer experiments, we further optimize the hidden dimension in the proteomics part of the network (from the values 16, 32 and 64) as it comprises two layers. The optimal hyperparameters for both sets of experiments are shown in Supplementary Tables 10 and 11.

Using these hyperparameters, we performed 25 iterations of each experiment using different train, test and validation splits. The training set is 70% of the data, and the test and validation sets are 15% each. We performed early stopping if the loss does not decrease in the test set for at least five consecutive epochs. To compute the final performance metrics, we averaged the predictions from the validation set across all the 25 iterations and used those averaged predictions to calculate the final prediction.

### Transformer-based architecture

We modified COMET to utilize a transformer-based architecture for learning and computing a latent representation from EHR data in lieu of embedding tokens with word2vec and using an RNN. We maintained the rest of the COMET architecture as described in the previous section for integrating the omics data.

All EHR data are preprocessed into sequential tokens. We include special tokens for the beginning of the sequence, and for marking the beginning and end of any given day. The maximum sequence length is 1,024, 6.8% of patients have sequences longer than this and their oldest EHR data are excluded. The network first maps each clinical code to a learned 128-dimension embedding vector. These embeddings are scaled by √d to preserve the magnitude during subsequent operations. To encode temporal information, we incorporate sinusoidal positional encodings PE(pos, 2*i*) = sin(pos/10,000^(2*i*/*d*)^) and PE(pos, 2*i* + 1) = cos(pos/10,000^(2*i*/*d*)^) for position pos and dimension *i*. These encodings are added to the scaled embeddings to produce position-aware input representations.

The core transformer uses two encoder layers, each using multihead self-attention with four heads. Following the attention mechanism, there is a two-layer feed-forward network. The first layer projects the intermediate representation into 512-dimensional space, applies a rectified linear unit nonlinearity followed by dropout and then projects it back to 128 dimensions. To handle variable-length sequences, we implement attention masking in which padding tokens are assigned large negative attention scores, effectively excluding them from attention computations. The final hidden state from the last position serves as the latent patient representation, which is used the same way as the RNN latent representation in the downstream parts of the network.

### Transformer-based architecture hyperparameter details

To determine the hyperparameters, we use a threefold cross-validation and grid search. Within the two training folds, we take 20% of the data as a validation set for early stopping. We assess the performance of each hyperparameter set via grid search on the validation set and choose the hyperparameter set that gives the lowest average loss on the validation sets across the threefold cross-validation. These hyperparameters are used for all subsequent experiments, including those with different train, test and validation splits. For pretraining the EHR part of the network, the parameter grid is as follows: learning_rate, {1 × 10^–^^2^, 1 × 10^–^^3^}; dropout, {0.1, 0.3}; lr_decay, {1 × 10^–^^2^, 1 × 10^–^^3^}; furthermore, the number of layers is fixed at 2; hidden dimension, fixed at 128; and batch size, fixed at 64. For the baseline omics experiments, we use the grid learning_rate of {1 × 10^–^^2^, 1 × 10^–^^3^, 1 × 10^–^^4^}, dropout of {0.1, 0.2, 0.3, 0.4, 0.5} and lr_decay of {1 × 10^–^^2^, 1 × 10^–^^3^, 1 × 10^–^^4^}; the number of layers is fixed at 2; hidden dimension, fixed at 128; and batch size, fixed at 16.

When we transfer the weights, we fix dropout as the optimal value used in the pretraining experiments. In the cancer experiments, the batch size is 32 for the pretraining cohort due to different hardware as those experiments are conducted in the UK Biobank’s Research Analysis Platform. We do not re-run the omics experiments as the only change in architecture is in the part of the network that analyses the EHR data. The optimal hyperparameters for the onset of labour experiments are shown in Supplementary Table 12 and for the cancer mortality experiments, in Supplementary Table 13.

Using these hyperparameters, we performed 25 iterations of each experiment using different train, test and validation splits. The training set is 70% of the data, and the test and validation sets are 15% each. We performed early stopping if the loss does not decrease in the test set for at least five consecutive epochs. To compute the final performance metrics, we averaged the predictions from the validation set across all 25 iterations and used those averaged predictions to calculate the final prediction.

### Ridge regression baseline

We implemented a ridge regression baseline, including an adaptation that can incorporate priors on the coefficients derived from pretraining. All features were preprocessed by removing low-variance features (threshold = 0.01) and applying standard normalization. The EHR features were one-hot encoded (that is, the feature value was 1 if the code occurred; otherwise, it was 0). For model selection, we employed threefold cross-validation with grouped splits by patient ID to prevent data leakage between related samples. The hyperparameter grid included regularization strengths of *λ* ∈ {0.1, 1, 5, 10, 25, 50, 75, 100, 250, 500, 1,000} and for the model incorporating a prior, prior strengths of *γ* ∈ {0, 0.25, 0.5, 0.75, 1}. The model minimizes the objective || *y* – *Xβ*||^2^ + *λ*||*β* – *γβ*_0_||^2^, where *n* is the sample size, *β*_0_ represents prior coefficients derived from pretraining and *γ* controls the influence of these priors. If the feature is not in the pretraining data (that is, all proteomics features), *β*_0_ is set to 0. The optimal hyperparameters were selected based on the minimum average r.m.s.e. across validation folds and are shown in Supplementary Table 14. For the final evaluation, predictions were generated using the held-out samples from cross-validation to ensure unbiased performance estimates.

### Logistic regression baseline

We implemented a logistic regression baseline, including an adaptation that can incorporate priors on the coefficients derived from pretraining. All features were preprocessed by removing low-variance features (threshold = 0.01) and applying standard normalization. The EHR features were one-hot encoded (that is, the feature value was 1 if the code occurred; otherwise, it was 0). For model selection, we employed threefold cross-validation with random splits. The hyperparameter grid included regularization strengths *C* ∈ {10⁻^8^, 10⁻^7^, 10⁻^6^, 10⁻^5^, 10⁻^4^, 10⁻^3^, 10⁻^2^} and for models incorporating priors, prior weights of *γ* ∈ {0, 0.25, 0.5, 0.75, 1}. The model minimizes the objective –log[*L*] + (1/2*C*)||*β* – *γβ*_0_||^2^, where *L* is the standard logistic likelihood function: $-{\rm{log}}({\prod}_{i}\,{p}({x_{i}})^{\;{y}_{i}}\,\times\,({1-p({x}_{i})})^{({1-Y}_{i})})$. Here *p*(*x*) = 1/(1 + e^(–*βx*)^), *β*_0_ represents prior coefficients derived from pretraining and *γ* controls the influence of these priors. Parameters were optimized using Newton–Raphson updates. The optimal hyperparameters were selected based on the maximum average AUROC across validation folds and are shown in Supplementary Table 15. For the final evaluation, predictions were generated using the held-out samples from cross-validation to ensure unbiased performance estimates.

### External validation

#### Onset of labour

We used an external dataset consisting of plasma proteomics from pregnant women to determine if the proteins identified as more important in the COMET models were more strongly correlated with days to the onset of labour in other patient cohorts^39^. We considered the 50 most important proteins among those that were more important in the COMET models (out of the 50, there were 14 proteins in the external dataset) and computed the Pearson correlation of these proteins with days to the onset of labour. We compared that with the Pearson correlation of the 50 most important proteins among those that were more important in the baseline models (out of the 50, there are 12 proteins in the external dataset).

#### Cancer mortality

We used an external dataset consisting of proteomics from breast cancer patients to determine if the proteins identified as more important in the COMET models were more strongly correlated with cancer mortality in other patient cohorts^40^. We considered the 50 most important proteins among those that were more important in the COMET models (out of the 50, there were 18 proteins in the external dataset) and computed the Pearson correlation of these proteins with days to the onset of labour. We compared that with the Pearson correlation of the 50 most important proteins among those that were more important in the baseline models (out of the 50, there are 18 proteins in the external dataset).

### Intermediate-node predictions

For each of the analyses that rely on intermediate representations from the EHR, protein and joint part of the network, we use the nodes just before the prediction head. These are represented by the light green (omics), light blue (EHR) and teal (joint) nodes that feed directly into the prediction head (Fig. 1c). The values in these nodes are a function of only the omics data, only the EHR data and both data modalities.

### Parameter-space visualization

It is difficult to visualize all the parameters of a complex neural network, and potentially not meaningful to do so as networks with different parameters can functionally be the same. Therefore, as others have done, we compare the function represented by each network, rather than comparing the parameters^7,41,42^. For each model (at each epoch), we input all the data points, compute the output and concatenate the outputs into a single vector (including the values at the intermediate node predictions as described above, which are used to visualize the protein, EHR and joint parameter space). The final output is used to visualize the overall parameter space. These vectors are concatenated into a single matrix, which is visualized in two dimensions using *t*-SNE.

### Reporting summary

Further information on research design is available in the Nature Portfolio Reporting Summary linked to this article.

## Supplementary information

Supplementary Information Supplementary Tables 1–15, Notes 1–5 and Figs. 1–14.

Reporting Summary

Supplementary Data Supplementary Data 1 and 2.

## Data Availability

The proteomics data for the pregnancy cohort are available at Dryad (http://datadryad.org/ and 10.5061/dryad.280gb5mpd). The EHR data for the pregnancy cohort cannot be shared publicly due to Stanford policies. The data (both proteomics and EHR) for the cancer mortality cohort are available through UK Biobank but cannot be shared publicly due to UK Biobank’s data use policies. The queries to pull the cohorts used in our study are included in the code at https://github.com/samson920/COMET/tree/main, and approved researchers with access to UK Biobank can replicate our analyses using these notebooks and the GitHub tutorial. The dataset used to externally validate the onset of labour feature importance are available at https://nalab.stanford.edu/multiomicsmulticohortpreterm/. The dataset used to externally validate the cancer mortality feature importance are available in the supplementary data of ref. ^40^.

## Code availability

All code is available at https://github.com/samson920/COMET/tree/main and via Zenodo (10.5281/zenodo.13977341)^43^. All the Stanford machine learning algorithms were trained on an NVIDIA Tesla P40, 24 GB GPU VRAM. All the UK Biobank machine learning models were trained within the UK Biobank Research Analysis Platform on an NVIDIA T4 GPU.
