# Supplementary material for: A machine learning approach to leveraging electronic health records for enhanced omics analysis
Source: Nat Mach Intell. 2025 Jan 16;7(2):293–306. doi: 10.1038/s42256-024-00974-9 (PMC11847705; doi:10.1038/s42256-024-00974-9)
Supplement: Supplementary file 1 — Supplementary Tables 1–15, Notes 1–5 and Figs. 1–14. [file 42256_2024_974_MOESM1_ESM.pdf]

nature machine intelligence

Supplementary information https://doi.org/10.1038/s42256-024-00974-9

A machine learning approach to leveraging electronic health records for enhanced omics analysis

In the format provided by the authors and unedited

Supplementary Table 1

	Lin's CCC
EHR Baseline	0.757
Proteomics Baseline	0.800
EHR + Proteomics Baseline	0.808
COMET	0.871

Agreement of COMET predictions for days to onset of labor with true outcomes.

Supplementary Table 2

Features	Pearson Correlation (95% CI)	RMSE
EHR Baseline	0.768 (0.685, 0.824)	20.4
Metabolomics Baseline	0.758 (0.678, 0.820)	21.1
EHR + Metabolomics Baseline	0.816 (0.753, 0.864)	18.7
COMET	**0.839 (0.782, 0.881)**	**18.1**

Performance of COMET for predicting days to onset of labor using EHR data and metabolomics data.

Supplementary Table 3

	Pearson R (95% CI)	RMSE
EHR Baseline	0.224 (0.076, 0.36)	31.3

Proteomics Baseline	0.628 (0.53, 0.71)	28.7
EHR + Proteomics Baseline	0.572 (0.46, 0.66)	29.6
EHR + Proteomics + Prior	0.799 (0.74, 0.85)	19.9

Performance of a ridge regression model for predicting days to onset of labor.

Supplementary Table 4

	COMET		COMET Transformer	
	Pearson R	RMSE	Pearson R	RMSE
Baseline EHR Only	0.768	20.4	0.686	23.2
Baseline EHR + Proteomics	0.815	18.4	0.818	18.5
COMET	**0.868**	**16.0**	**0.848**	**17.0**

Comparison of COMET to a variation that utilizes a transformer instead of an RNN to learn a latent representation of EHR data.

Supplementary Table 5

	Cohen's Kappa
EHR Baseline	0.045
Proteomics Baseline	0.210
EHR + Proteomics Baseline	0.202
COMET	0.458

Agreement of COMET predictions for cancer mortality with true outcomes using 0.5 as the threshold to classify predictions.

Supplementary Table 6

	AUROC (95% CI)	AUPRC (95% CI)

EHR Baseline	0.744 (0.70, 0.78)	0.118 (0.04, 0.20)
Proteomics Baseline	0.823 (0.79, 0.85)	0.263 (0.21, 0.36)
EHR + Proteomics Baseline	0.832 (0.80, 0.86)	0.263 (0.18, 0.34)
EHR + Proteomics + Prior	0.841 (0.81, 0.86)	0.279 (0.20, 0.35)

Performance of logistic regression models for predicting cancer mortality.

Supplementary Table 7

	COMET		COMET Transformer	
	AUROC	AUPRC	AUROC	AUPRC
Baseline EHR Only	0.749	0.205	0.802	0.280
Baseline EHR + Proteomics	0.786	0.365	0.812	0.423
COMET	**0.842**	**0.504**	**0.833**	**0.452**

Comparison of COMET to a variation that utilizes a transformer instead of an RNN to learn a latent representation of EHR data.

Supplementary Table 8: Demographics for Pregnancy Cohorts

	Pre-Training Cohort	Omics Cohort
Mean Age at Birth (SD)	32.1 (5.7)	32.2 (3.2)
Baby Sex Male	15980 (51.8%)	37 (60.7%)
Baby Sex Female	14659 (47.5%)	24 (39.3%)
Baby Sex Unknown	204 (0.7%)	0 (0.0%)
Race - White	9545 (31.0%)	20 (32.8%)
Race - Asian	7768 (25.2%)	25 (41.0%)
Race - Black or African American	644 (2.1%)	0 (0.0%)
Race - Native Hawaiian or	469 (1.5%)	1 (1.6%)

Other Pacific Islander		
Race - American Indian or Alaska Native	40 (0.1%)	0 (0.0%)
Race - Other	10831 (35.1%)	9 (14.8%)
Race - Unknown	1236 (4.0%)	6 (9.8%)
Ethnicity - Hispanic or Latino	9855 (32.0%)	5 (8.2%)
Ethnicity - Not Hispanic or Latino	20485 (66.4%)	54 (88.5%)
Ethnicity - Unknown	503 (1.6%)	2 (3.3%)

23

Supplementary Table 9: Demographics for Cancer Mortality Cohorts

	Pre-Training Cohort	Omics Cohort
Mean Age at Diagnosis (SD)	62.1 (7.5)	59.2 (7.7)
Sex Male	18792 (50.9%)	305 (54.6%)
Sex Female	18109 (49.1%)	254 (45.4%)
Race - White	35052 (94.9%)	528 (94.5%)
Race - Asian Indian	450 (1.2%)	6 (1.1%)
Race - Asian	116 (0.3%)	1 (0.2%)
Race - African	178 (0.5%)	10 (1.8%)
Race - Pakistani	132 (0.4%)	3 (0.5%)
Race - Chinese	76 (0.2%)	0 (0.0%)
Race - Bangladeshi	10 (0.03%)	0 (0.0%)
Race - Black	5 (0.01%)	0 (0.0%)
Race - Unknown	902 (2.4%)	11 (2.0%)
Ethnicity - Unknown	36901 (0.0%)	559 (100%)

Supplementary Table 10

	Learning Rate	Dropout	Learning Rate Decay	Layers
EHR Baseline	0.0001	0.1	0.001	4
Proteomics Baseline	0.001	N/A	0.1	N/A
EHR + Proteomics Baseline	0.0001	0.3	0.1	4
COMET	0.01	0.5	0.0001	2

Optimal hyperparameters for predicting days to onset of labor.

Supplementary Table 11

	Learning Rate	Dropout	Learning Rate Decay	Layers	Proteomics Hidden Dim
EHR Baseline	0.001	0.1	0.0001	4	N/A
Proteomics Baseline	0.001	N/A	0.01	N/A	64
EHR + Proteomics Baseline	0.01	0.5	0.01	4	64
COMET	0.01	0.1	0.01	2	64

Optimal hyperparameters for predicting days cancer mortality

Supplementary Table 12

	Learning Rate	Dropout	Learning Rate Decay
EHR Baseline	0.001	0.3	0.0001
EHR + Proteomics Baseline	0.01	0.1	0.01
COMET Transformer	0.01	0.1	0.01

Optimal hyperparameters for COMET transformer experiments for predicting days to onset of labor.

Supplementary Table 13

	Learning Rate	Dropout	Learning Rate Decay
EHR Baseline	0.001	0.3	0.001
EHR + Proteomics Baseline	0.001	0.3	0.01
COMET Transformer	0.001	0.3	0.001

Optimal hyperparameters for COMET transformer experiments for predicting days to onset of labor.

Supplementary Table 14

	Lambda	Gamma
EHR Baseline	250	N/A
Proteomics Baseline	100	N/A
EHR + Proteomics Baseline	250	N/A
EHR + Proteomics + Prior	500	1

Optimal hyperparameters for ridge regression experiments.

Supplementary Table 15

	C	Gamma
EHR Baseline	10^{-4}	N/A
Proteomics Baseline	10^{-3}	N/A
EHR + Proteomics Baseline	10^{-4}	N/A
EHR + Proteomics + Prior	10^{-4}	0.5

Optimal hyperparameters for logistic regression experiments.

Supplementary Note 1

sST2 plays an important role in immune regulation during pregnancy, is known to vary throughout pregnancy progression, and is associated with the onset of several pregnancy complications such as preeclampsia and preterm labor.[24,25] Cystatin C has also been shown to be a prognostic factor for gestational complications, and is especially relevant in preeclampsia in the context of compromised renal function.[26,27] PLXB2 is a fetal membrane / endometrial protein, which has been shown to play a role in embryo attachment and vary throughout pregnancy.[28,44]

In the baseline experiments, the most correlated proteins were sICAM-3, LRRT1, and angiopoietin-4. The role of sICAM-3 has not been investigated in pregnancy, though one study compared levels of sICAM-3 in women ten years after they had severe early onset pre-eclampsia and found that they were not different from controls with uncomplicated pregnancies.[45] LRRT1 also does not have a known role in pregnancy and is better known for its role in organizing synapse development.[46] While the expression of angiopoietin-4 does vary throughout pregnancy in rat placenta, it is not as well studied as angiopoietin-2, one of the top features for predicting the time to labor in the original study[21], and for which there is human genetics-based evidence associating it with preterm labor.[47,48] Angiopoietin-2 was one the top 10 proteins with the greatest number of significant correlations with the EHR latent representation dimensions in the COMET models, but not in the baseline models, further suggesting a meaningful alignment occurs in the COMET models.

Supplementary Note 2

Many of the proteins that gained importance with COMET are known to play a role in pregnancy progression, fetal development, or pregnancy complications that have implications for labor timing. For example, SPINT2 is elevated in the placenta of patients who required preterm delivery due to preeclampsia.[49] DDR1 has been implicated in birth timing and associated with gestational age.[21,50,51] VEGFR sR3 plasma levels have been shown to change throughout human pregnancy.[52] MMP12 plays a major role in remodeling of spiral arteries, which allows for proper placental development and maternal blood flow to the fetus; irregularities in spiral artery remodeling can cause fetal and maternal stress associated with preeclampsia.[53,54] Conversely, the proteins that became less important with COMET have other biological functions. SLAF6 is a co-receptor for natural killer (NK) cell activation, and plays a role in T cell exhaustion with no known role in pregnancy.[55] DRG1 is involved in mitotic spindle assembly and GTPase activity, and is primarily known to play a role in tumor metastasis across cancers.[56,57]

Supplementary Note 3

PGF is actively being investigated as a novel anti-cancer target, and has been shown to be a prognostic biomarker for mortality risk across several cancers.[58] EDA2R is a transmembrane protein in the tumor necrosis factor receptor family that has been associated with cancer cachexia across multiple cancers and has been identified as a prognostic factor in both prostate cancer and breast cancer.[59-61] GDF15 is a member of the glial cell-derived neurotropic factor family with generally very low levels in healthy humans, but has been associated with many diseases and is considered a biomarker for all-cause mortality across these diseases, including cancer.[62,63]

Supplementary Note 4

Carcinoembryonic antigen-related cell adhesion molecule 5 (CEACAM5) is a prognostic biomarker that stimulates tumor proliferation in pancreatic, colon, and non-small cell lung cancers.[64-66] Cytokeratin 19 (KRT19) plays a role in reprogramming cancer stem-cell like cells to be less aggressive and more drug sensitive, and is a prognostic biomarker in breast and prostate cancer.[67-69] Syndecan 1 (SDC1) plays a role in tumor microenvironment modulation and is a prognostic biomarker associated with cancer metastasis and drug resistance.[70]

Supplementary Note 5

Here we write out a proof showing that the weight updates in the proteomics part of the network are a function of the EHR weights that are learned via pre-training.

Notation

- $\mathbf{W}^{(t)}_{\text{proteomics}}$: Weights of the proteomics layer at time step t.
- $\mathbf{W}^{(t)}_{\text{GRU}}$: Weights of the GRU layer at time step t.
- η: Learning rate.
- y: True labels.
- $\hat{y}$: Predicted labels (output of the model).
- L: Loss function.
- $\mathbf{x}_{\text{proteomics}}$: Input to the proteomics layer.
- $\mathbf{h}_{\text{GRU}}$: Output of the GRU layer.
- $\hat{y}_{\text{proteomics}}$: Predicted output from the proteomics part of the network.
- $\hat{y}_{\text{EHR}}$: Predicted output from the EHR part of the network.
- $\hat{y}_{\text{joint}}$: Predicted output from the joint part of the network.
- $w_{\text{final,proteomics}}$: Weight for the proteomics output in the final layer of the network.

The weight update formula for the proteomics layer is given by:

$$\mathbf{W}^{(t+1)}_{\text{proteomics}} = \mathbf{W}^{(t)}_{\text{proteomics}} - \eta \nabla_{\mathbf{W}^{(t)}_{\text{proteomics}}} L$$

Using the chain rule, the gradient $\nabla_{\mathbf{W}^{(t)}_{\text{proteomics}}} L$ can be expressed as:

$$\nabla_{\mathbf{W}^{(t)}_{\text{proteomics}}} L = \frac{\partial L}{\partial \hat{y}} \cdot \frac{\partial \hat{y}}{\partial \hat{y}_{\text{proteomics}}} \cdot \frac{\partial \hat{y}_{\text{proteomics}}}{\partial \mathbf{W}^{(t)}_{\text{proteomics}}}$$

Gradient of Loss with Respect to Predicted Labels:

$$\frac{\partial L}{\partial \hat{y}} = \frac{2}{n}(\hat{y} - y)$$

Gradient of Final Prediction with Respect to Predicted Proteomics:

$$\frac{\partial \hat{y}}{\partial \hat{y}_{\text{proteomics}}} = w_{\text{final,proteomics}}$$

Gradient of Predicted Proteomics with Respect to Proteomics Weights:

$$\frac{\partial \hat{y}_{\text{proteomics}}}{\partial \mathbf{W}_{\text{proteomics}}^{(t)}} = \mathbf{x}_{\text{proteomics}}^{T}$$

Substitute the Components into the Gradient Formula:

$$\nabla_{\mathbf{W}_{\text{proteomics}}^{(t)}} L = \left(\frac{2}{n}(\hat{y} - y)\right) \cdot w_{\text{final,proteomics}} \cdot \mathbf{x}_{\text{proteomics}}^{T}$$

Substitute Into Weight Update for Proteomics:

$$\mathbf{W}_{\text{proteomics}}^{(t+1)} = \mathbf{W}_{\text{proteomics}}^{(t)} - \eta\left(\frac{2}{n}(\hat{y} - y) \cdot w_{\text{final,proteomics}} \cdot \mathbf{x}_{\text{proteomics}}^{T}\right)$$

Influence of GRU Weights:

The predicted label $\hat{y}$ depends on:

- $\hat{y}_{\text{proteomics}} = \mathbf{W}_{\text{proteomics}}\mathbf{x}_{\text{proteomics}}$
- $\hat{y}_{\text{EHR}} = \mathbf{W}_{\text{EHR}}\mathbf{h}_{\text{GRU}}$
- $\hat{y}_{\text{joint}} = \mathbf{W}_{\text{joint}}(\mathbf{h}_{\text{GRU}} \parallel \mathbf{x}_{\text{proteomics}})$

Here, $\mathbf{h}_{\text{GRU}}$ is a function of the GRU weights $\mathbf{W}_{\text{GRU}}^{(t)}$. Therefore, $\hat{y}$ depends on $\mathbf{h}_{\text{GRU}}$, which in turn depends on $\mathbf{W}_{\text{GRU}}^{(t)}$.

Conclusion:

Since the gradient $\nabla_{\mathbf{W}_{\text{proteomics}}^{(t)}} L$ includes $\hat{y}$, and $\hat{y}$ is influenced by $\mathbf{h}_{\text{GRU}}$, which depends on $\mathbf{W}_{\text{GRU}}^{(t)}$, the weight update $\mathbf{W}_{\text{proteomics}}^{(t+1)}$ is a function of $\mathbf{W}_{\text{GRU}}^{(t)}$.

Therefore, we can conclude that the weight update in the proteomics layer is indeed influenced by the weights in the EHR part of the network which are learned via pre-training.

Supplementary Figures

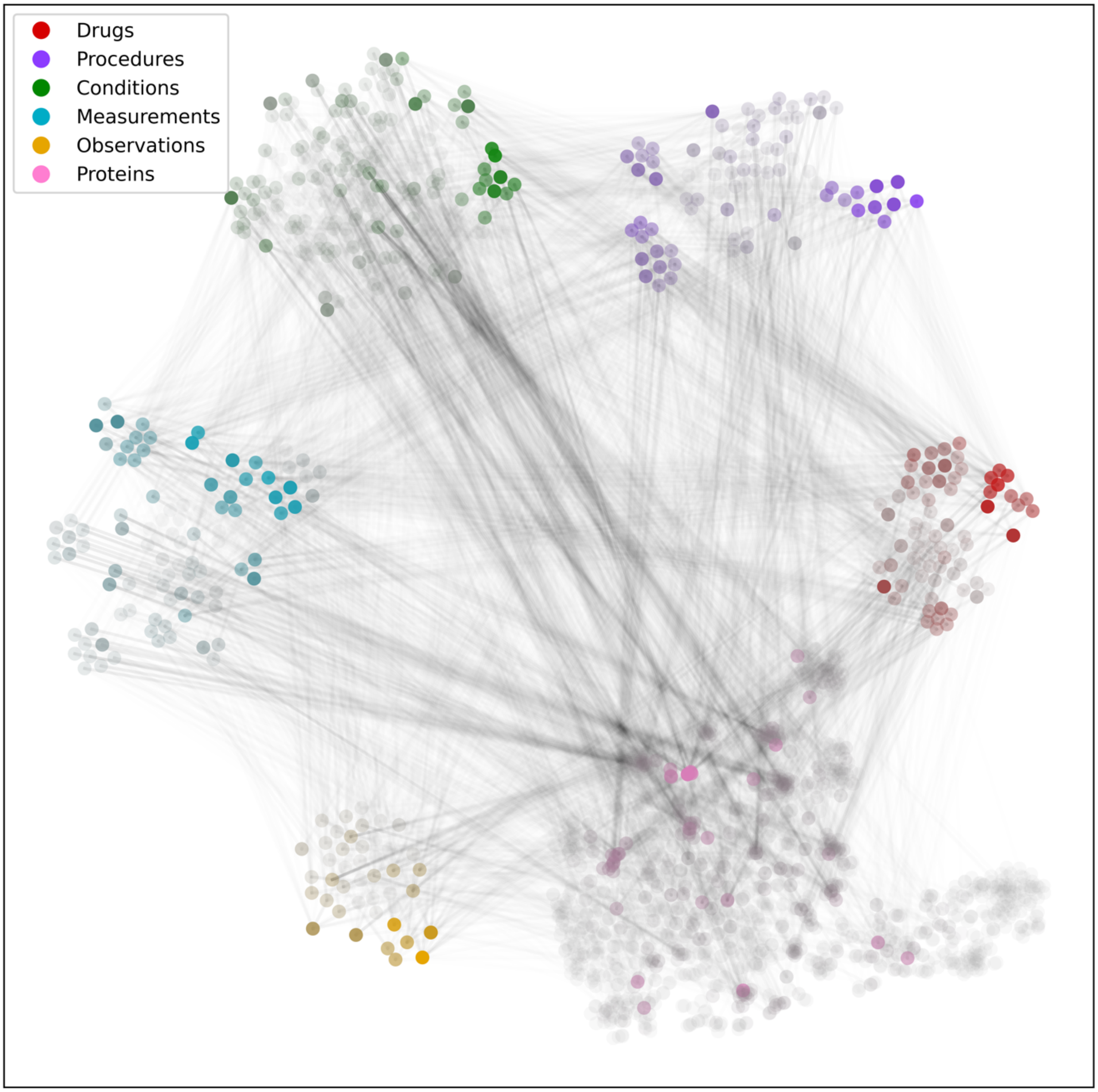

Supplementary Figure 1: Correlation network of the onset of labor dataset by modality. A 2-dimensional representation for each modality is learned separately via t-SNE, lines represent significant correlations of variables across modalities. The shade of the nodes represents the number of significant correlations they have with features in other modalities. Faint gray nodes have few significant correlations, and colored nodes have a high number of significant correlations. Some proteins are significantly correlated with many EHR variables, suggesting overlapping information, but others have no significant correlations with any EHR variables, suggesting complementary information.

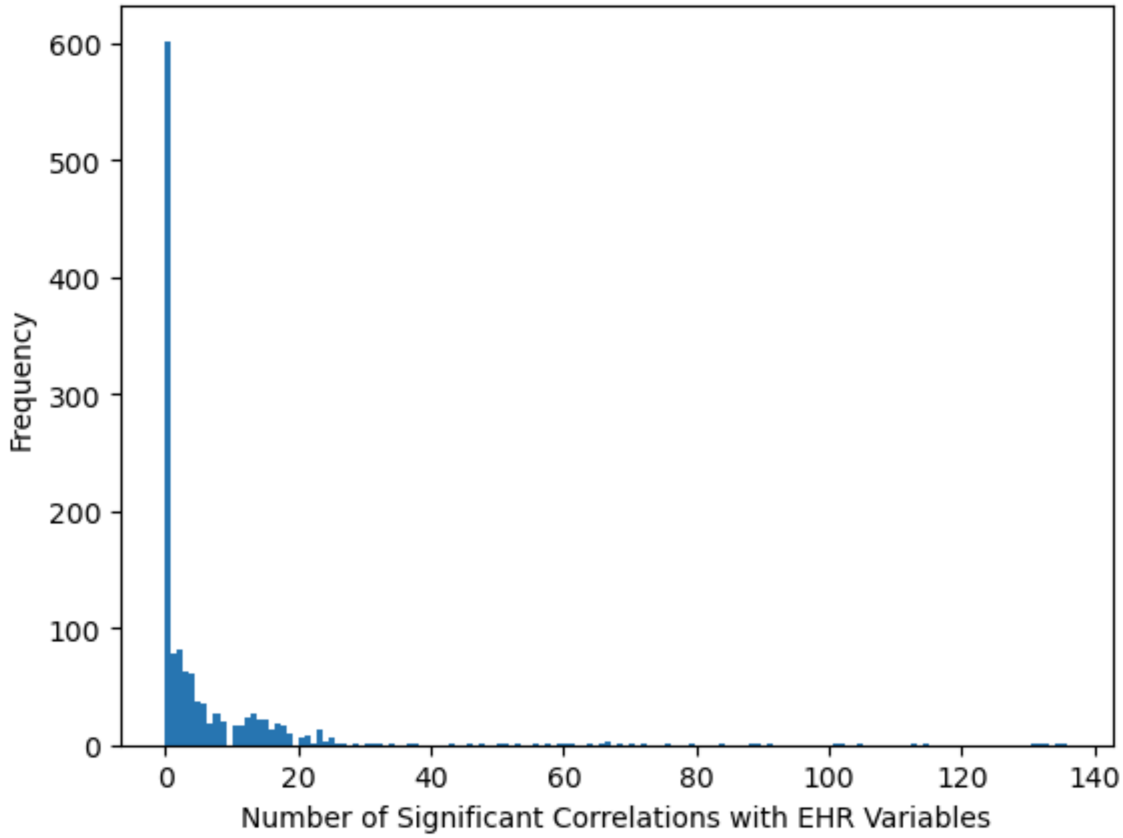

Supplementary Figure 2: Distribution of number of significant correlations that each protein variable has with all EHR variables in the onset of labor dataset. 46.1% of the proteins have no significant correlation with any EHR variable, suggesting they provide novel information about the patient's physiological state.

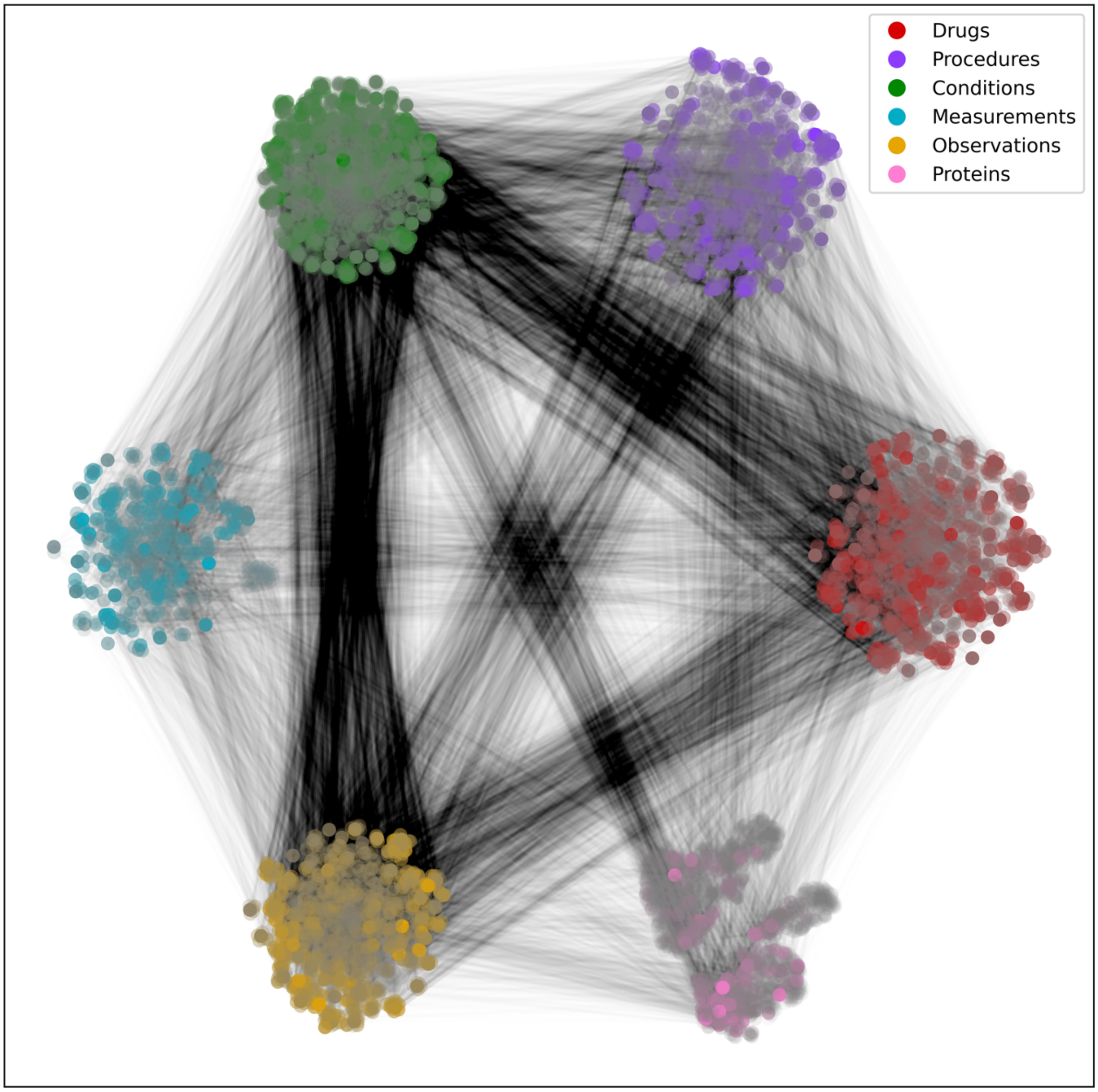

Supplementary Figure 3: Correlation network of the onset of cancer mortality dataset by modality. A 2-dimensional representation for each modality is learned separately via t-SNE, lines represent significant correlations of variables across modalities. The shade of the nodes represents the number of significant correlations they have with features in other modalities. Faint gray nodes have few significant correlations, and colored nodes have a high number of significant correlations. Some proteins are significantly correlated with many EHR variables, suggesting overlapping information, but others have no significant correlations with any EHR variables, suggesting complementary information.

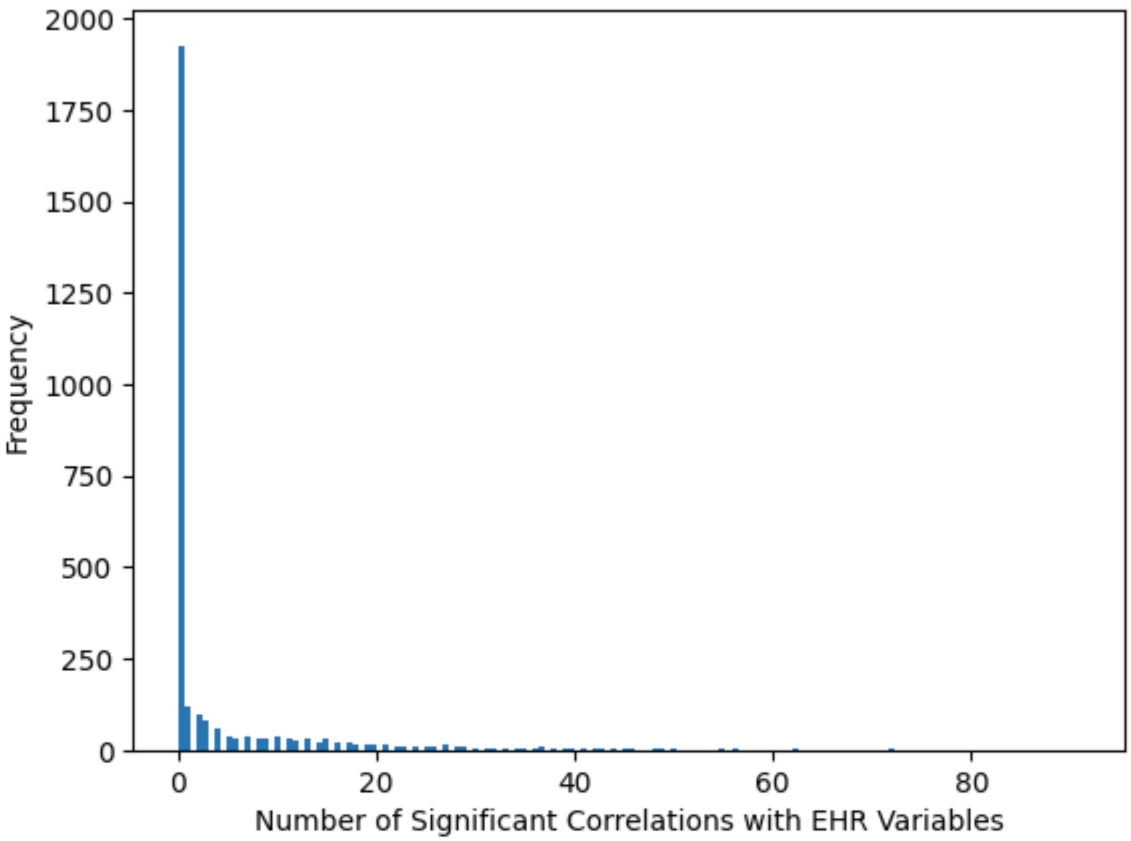

Supplementary Figure 4: Distribution of number of significant correlations that each protein variable has with all EHR variables in the cancer mortality dataset. 65.9% of the proteins have no significant correlation with any EHR variable, suggesting they provide information about the patient’s physiological state that is not captured in the EHR data.

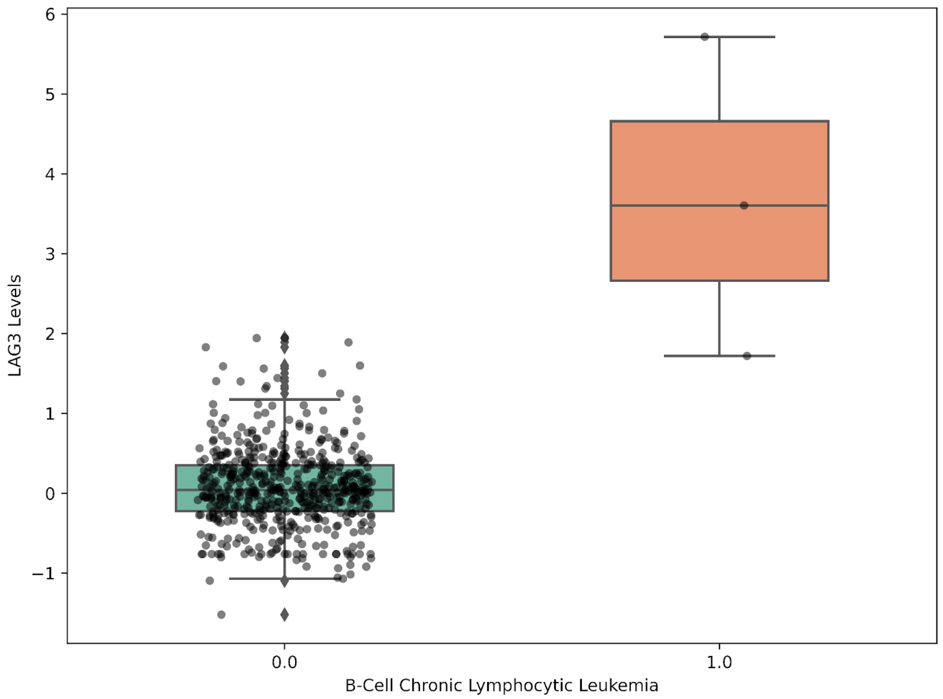

Supplementary Figure 5: LAG3 protein intensity for patients with and without a diagnosis of B-cell chronic lymphocytic leukemia (n = 3). Box plots show the median (center line), 25th and 75th percentiles (box bounds), with whiskers extending to the most extreme data points within 1.5 times the interquartile range from the box edges.

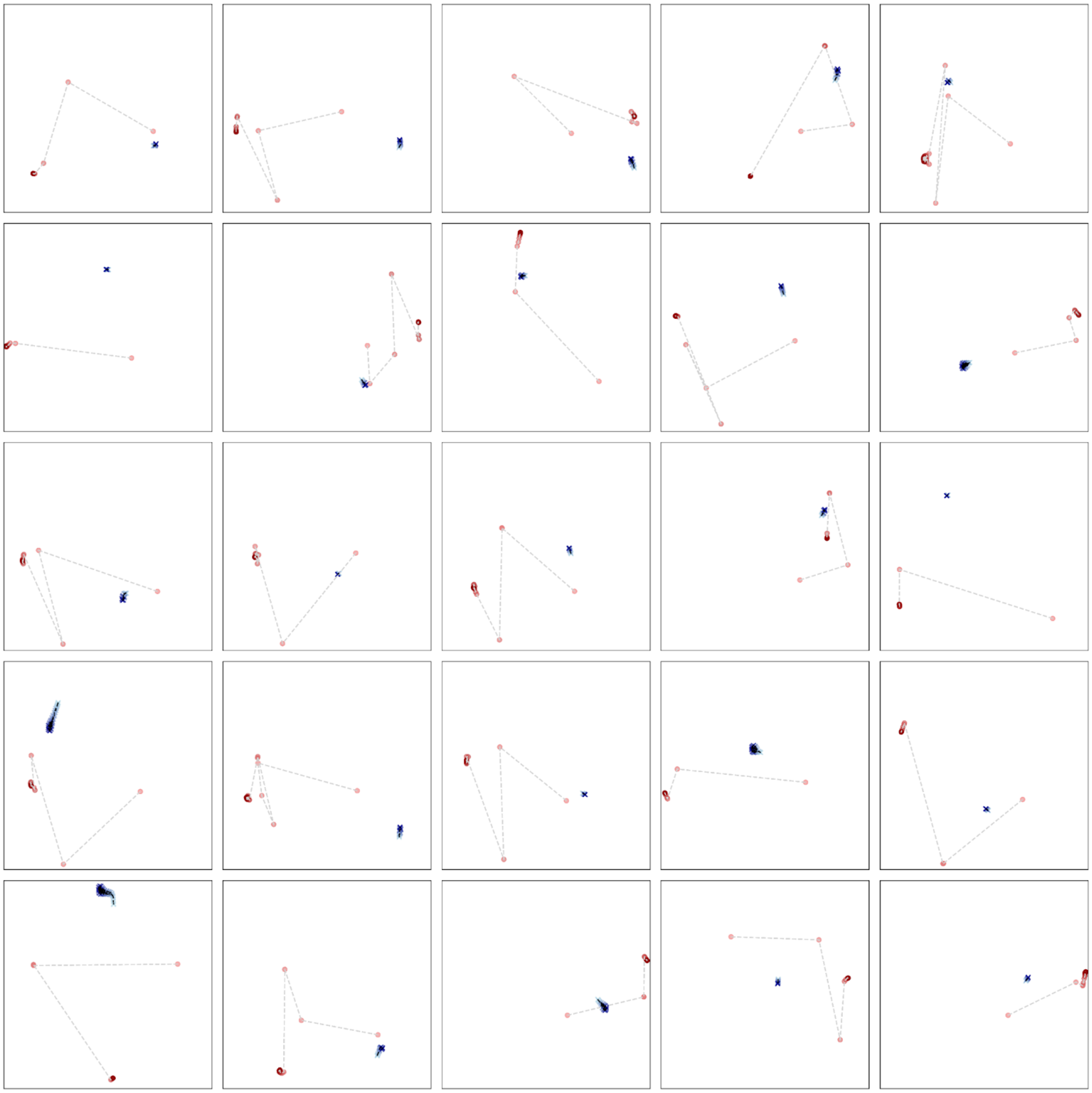

Supplementary Figure 6: Visualization of the protein-only parameters moving through the parameter space during training of the onset of labor models. Each of the 25 iterations is visualized separately. Red circles with gray lines represent COMET, and blue X's with black lines represent the joint baseline model. In each iteration of the experiments, the COMET model converges to a different part of the parameter space which is not visited by the baseline model, suggesting that COMET allows the model to converge to sets of parameters (which result in better performing models) that are not possible with existing methods).

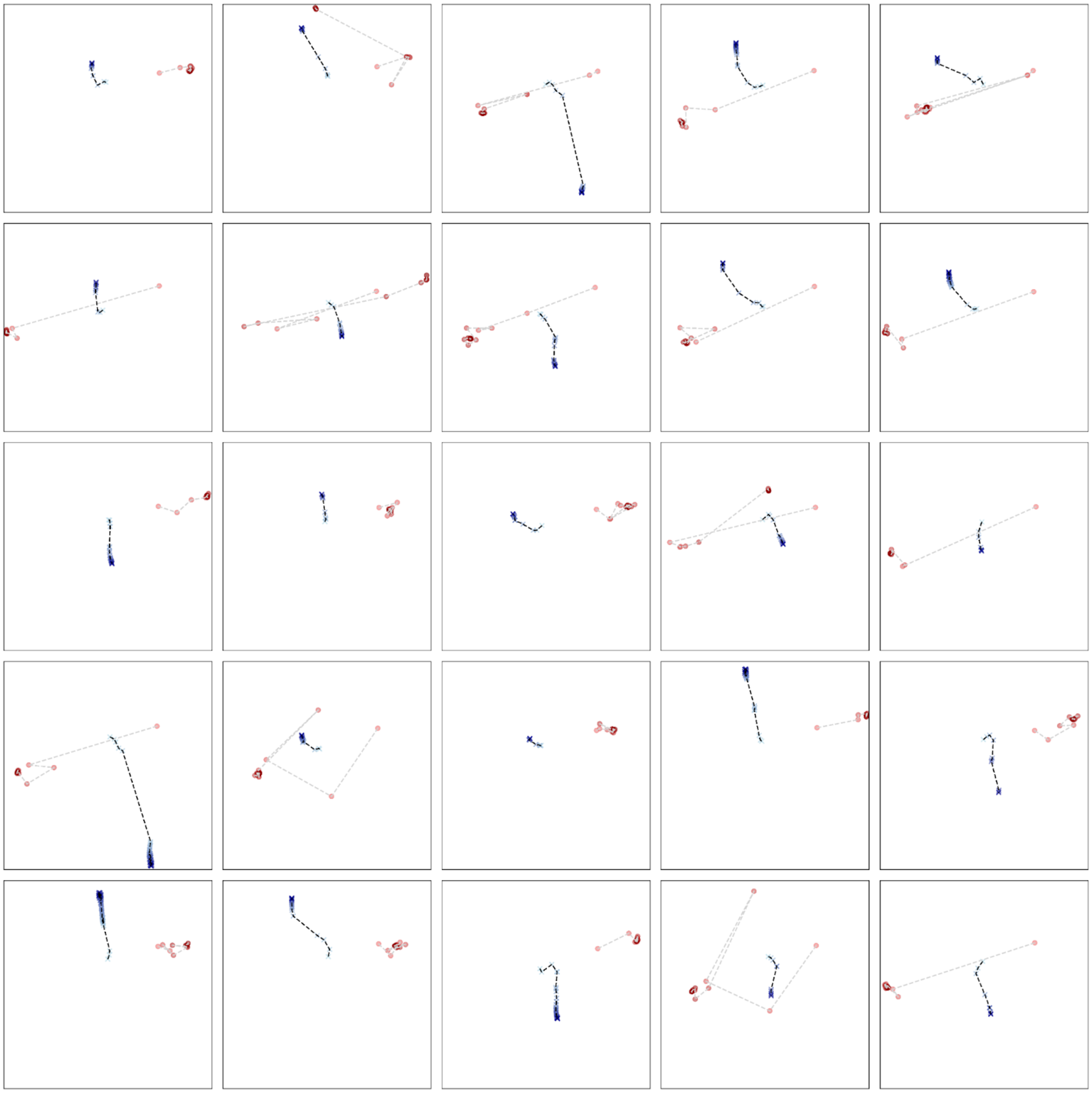

Supplementary Figure 7: Visualization of the EHR-only parameters moving through the parameter space during training of the onset of labor models. Each of the 25 iterations is visualized separately. Red circles with gray lines represent COMET, and blue X’s with black lines represent the joint baseline model. In each iteration of the experiments, the COMET model converges to a different part of the parameter space which is not visited by the baseline model, suggesting that COMET allows the model to converge to sets of parameters (which result in better performing models) that are not possible with existing methods).

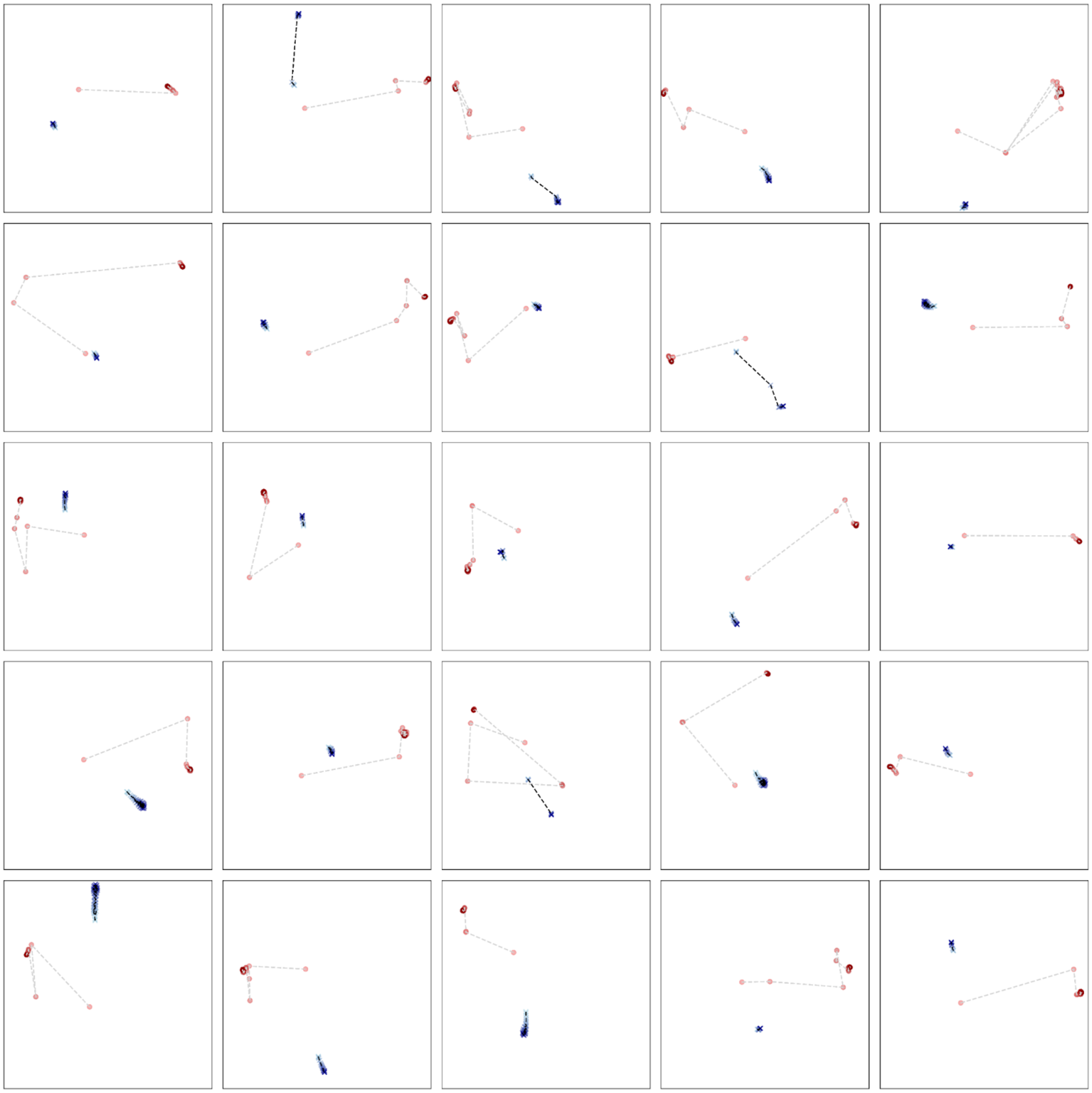

Supplementary Figure 8: Visualization of the joint parameters moving through the parameter space during training of the onset of labor models. Each of the 25 iterations is visualized separately. Red circles with gray lines represent COMET, and blue X's with black lines represent the joint baseline model. In each iteration of the experiments, the COMET model converges to a different part of the parameter space which is not visited by the baseline model, suggesting that COMET allows the model to converge to sets of parameters (which result in better performing models) that are not possible with existing methods).

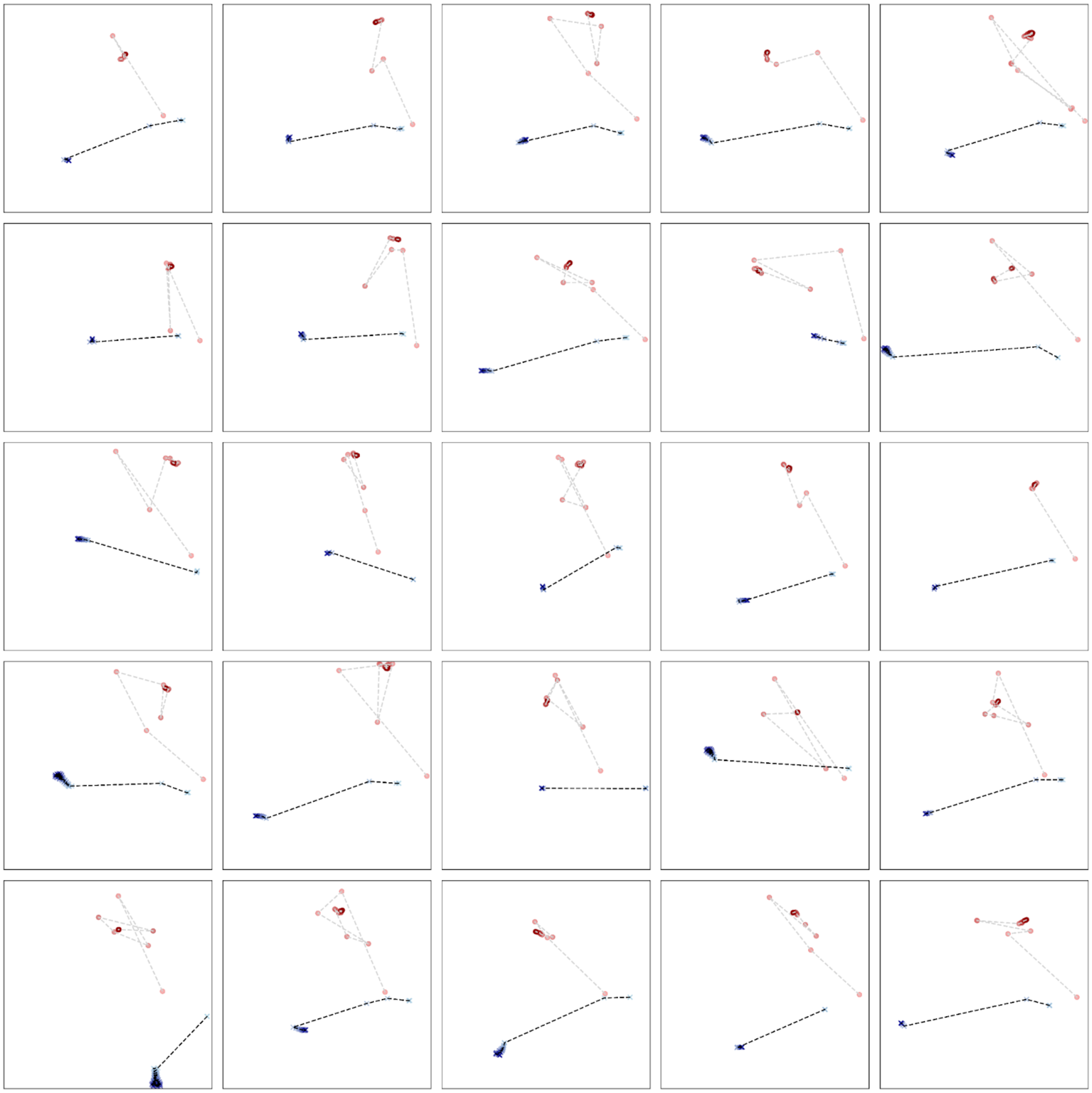

Supplementary Figure 9: Visualization of the overall parameters moving through the parameter space during training of the onset of labor models. Each of the 25 iterations is visualized separately. Red circles with gray lines represent COMET, and blue X's with black lines represent the joint baseline model. In each iteration of the experiments, the COMET model converges to a different part of the parameter space which is not visited by the baseline model, suggesting that COMET allows the model to converge to sets of parameters (which result in better performing models) that are not possible with existing methods).

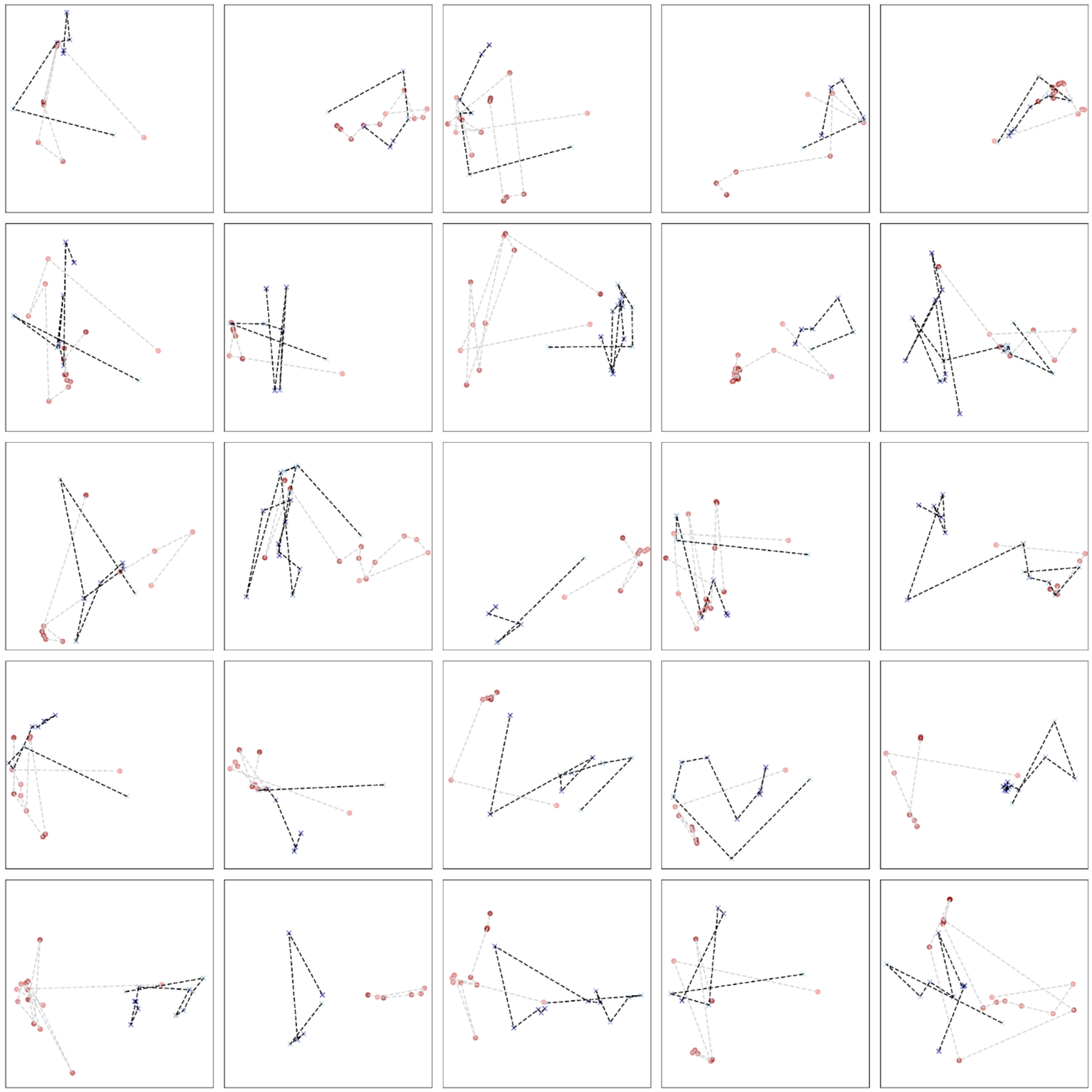

Supplementary Figure 10: Visualization of the protein-only parameters moving through the parameter space during training of the cancer mortality models. Each of the 25 iterations is visualized separately. Red circles with gray lines represent COMET, and blue X's with black lines represent the joint baseline model. In each iteration of the experiments, the COMET model converges to a different part of the parameter space which is not visited by the baseline model, suggesting that COMET allows the model to converge to sets of parameters (which result in better performing models) that are not possible with existing methods).

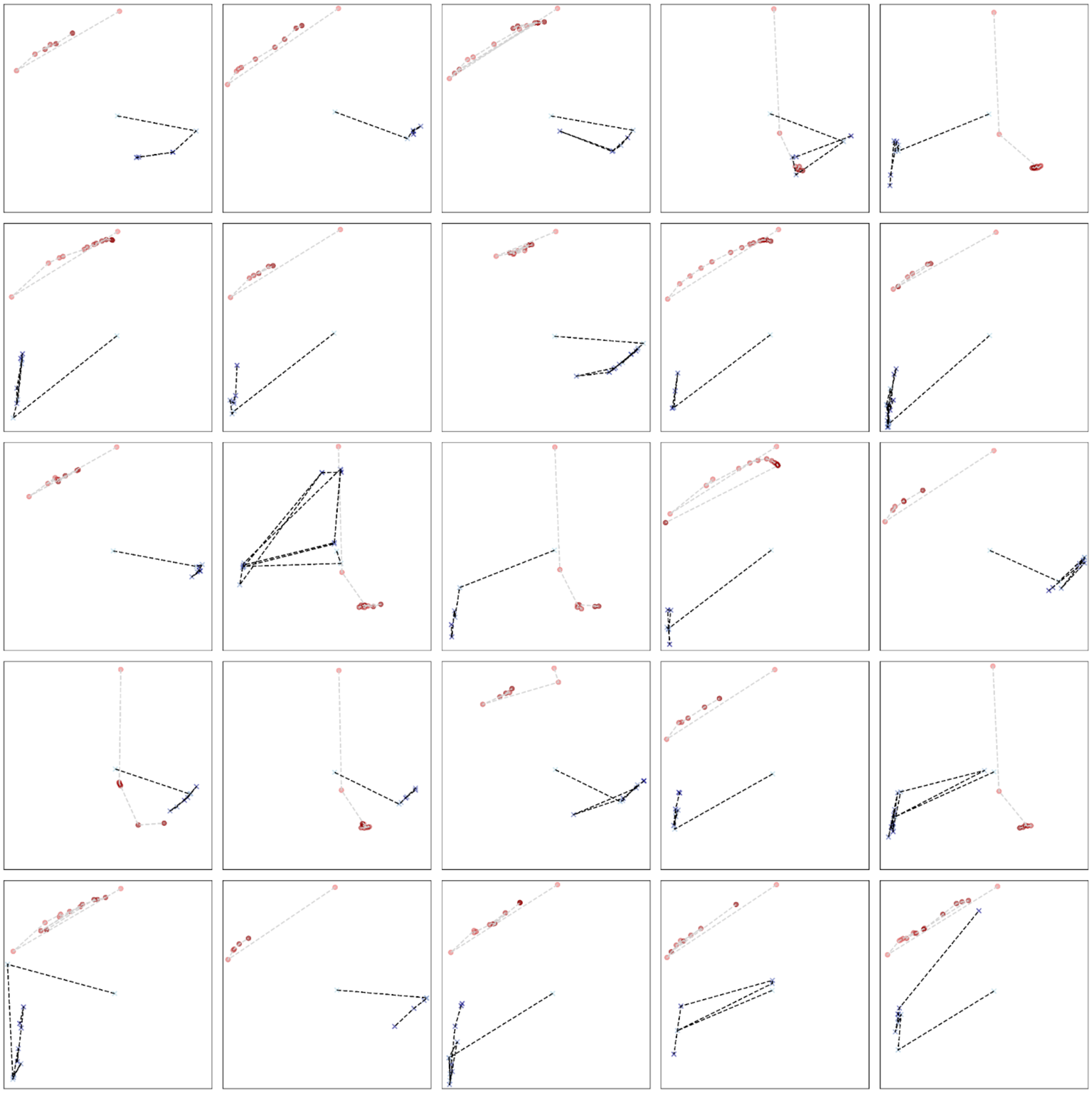

Supplementary Figure 11: Visualization of the EHR-only parameters moving through the parameter space during training of the cancer mortality models. Each of the 25 iterations is visualized separately. Red circles with gray lines represent COMET, and blue X's with black lines represent the joint baseline model. In each iteration of the experiments, the COMET model converges to a different part of the parameter space which is not visited by the baseline model, suggesting that COMET allows the model to converge to sets of parameters (which result in better performing models) that are not possible with existing methods).

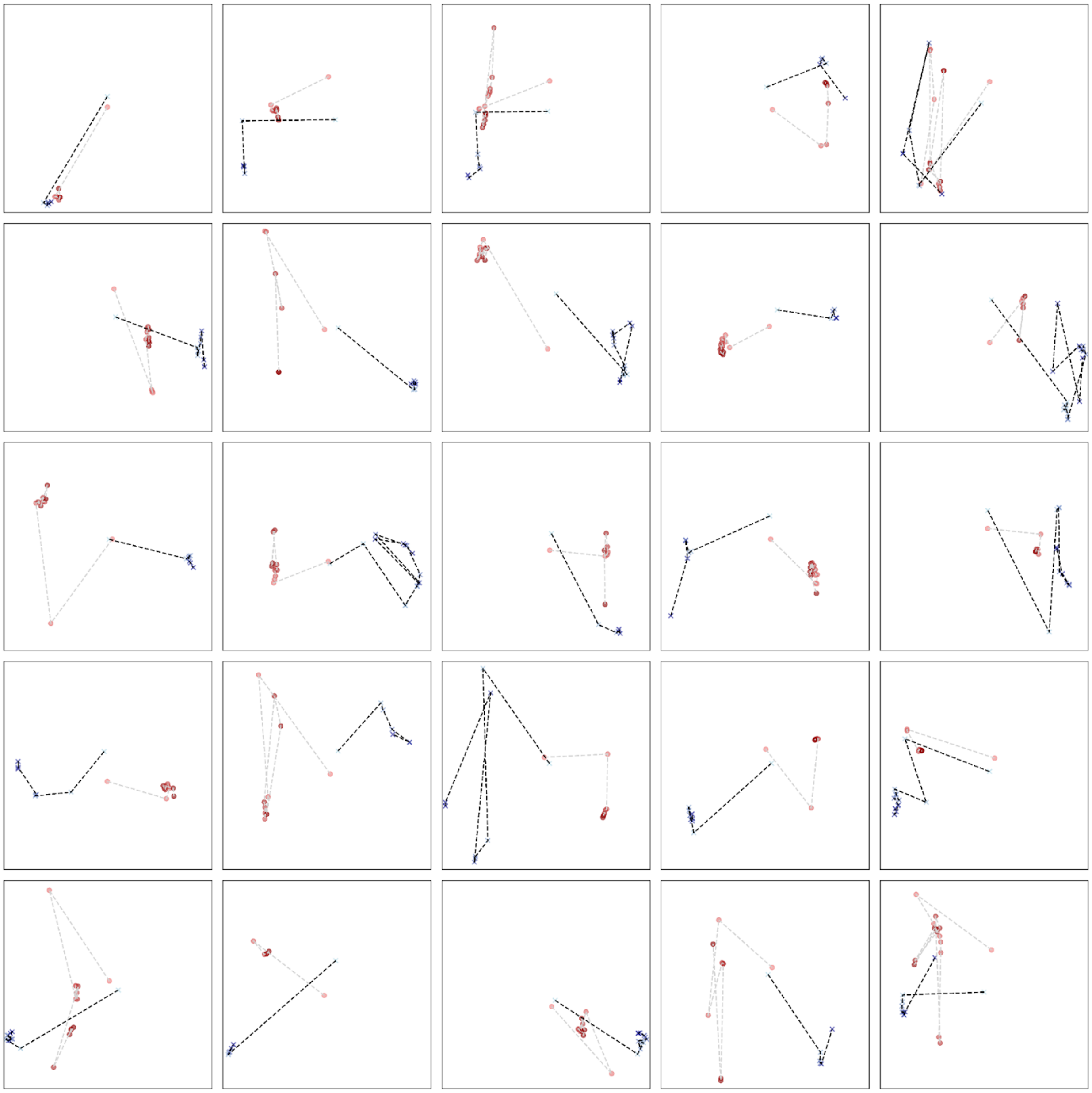

Supplementary Figure 12: Visualization of the joint parameters moving through the parameter space during training of the cancer mortality models. Each of the 25 iterations is visualized separately. Red circles with gray lines represent COMET, and blue X's with black lines represent the joint baseline model. In each iteration of the experiments, the COMET model converges to a different part of the parameter space which is not visited by the baseline model, suggesting that COMET allows the model to converge to sets of parameters (which result in better performing models) that are not possible with existing methods).

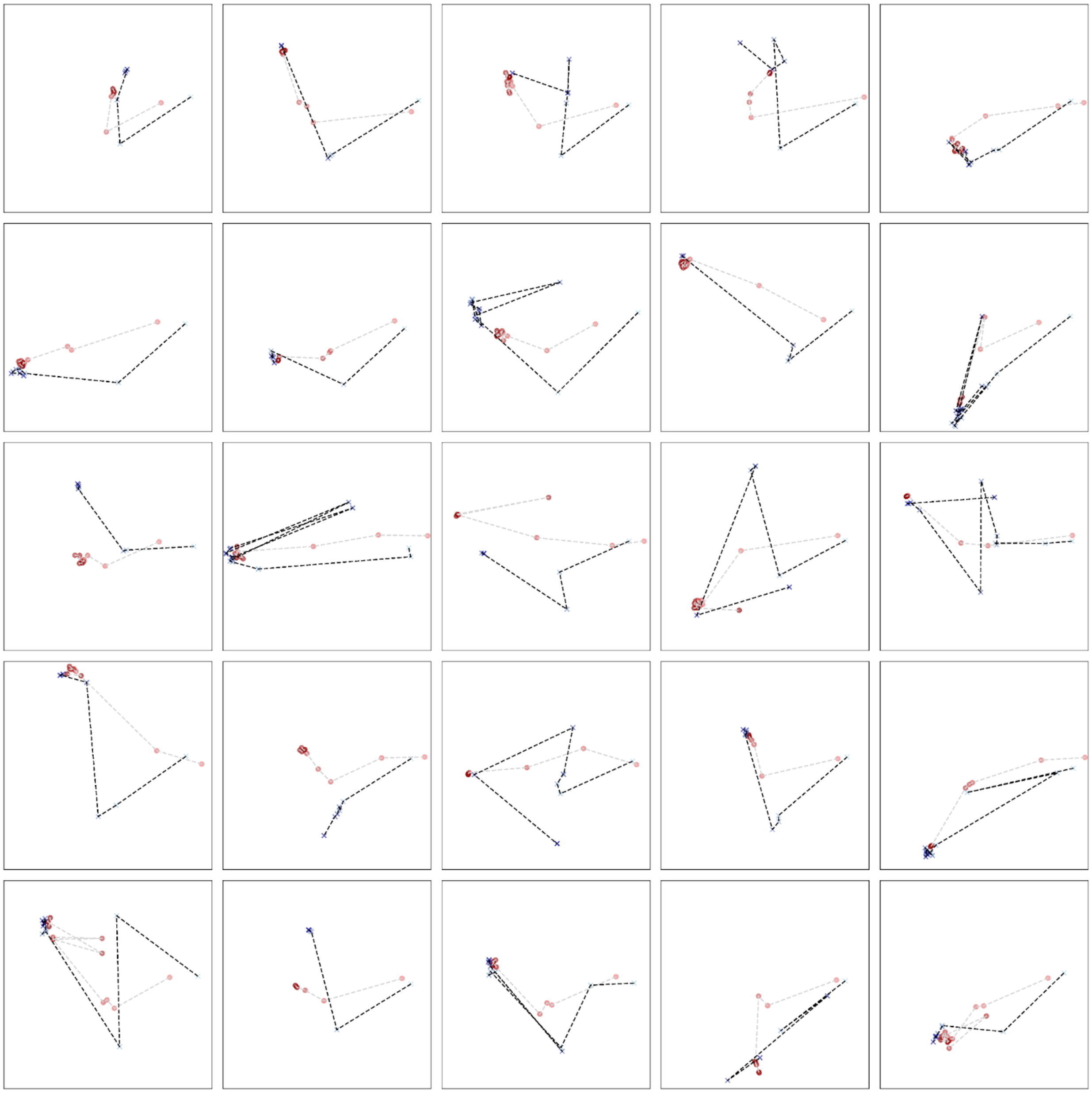

Supplementary Figure 13: Visualization of the overall parameters moving through the parameter space during training of the cancer mortality models. Each of the 25 iterations is visualized separately. Red circles with gray lines represent COMET, and blue X's with black lines represent the joint baseline model. In each iteration of the experiments, the COMET model converges to a different part of the parameter space which is not visited by the baseline model, suggesting that COMET allows the model to converge to sets of parameters (which result in better performing models) that are not possible with existing methods).

44.Aghaeepour, N. et al. A proteomic clock of human pregnancy. Am. J. Obstet. Gynecol. 218, 347.e1-347.e14 (2018).

45.Gaugler-Senden, I. P. M. et al. Angiogenic factors in women ten years after severe very early onset preeclampsia. PLoS ONE 7, e43637 (2012).

46.de Arce, K. P. et al. Concerted roles of LRRTM1 and SynCAM 1 in organizing prefrontal cortex synapses and cognitive functions. Nat. Commun. 14, 459 (2023).

47.Vaswani, K. et al. The effect of gestational age on angiogenic gene expression in the rat placenta. PLoS ONE 8, e83762 (2013).

48.Wujcicka, W. I. et al. Association of Single Nucleotide Polymorphisms from Angiogenesis-Related Genes, ANGPT2, TLR2 and TLR9, with Spontaneous Preterm Labor. Curr. Issues Mol. Biol. 44, 2939–2955 (2022).

49.Murphy, C. N. et al. Elevated Circulating and Placental SPINT2 Is Associated with Placental Dysfunction. Int. J. Mol. Sci. 22, (2021).

50.Apps, R. et al. Multimodal immune phenotyping of maternal peripheral blood in normal human pregnancy. JCI Insight 5, (2020).

51.Plunkett, J. et al. An evolutionary genomic approach to identify genes involved in human birth timing. PLoS Genet. 7, e1001365 (2011).

52.Romero, R. et al. The maternal plasma proteome changes as a function of gestational age in normal pregnancy: a longitudinal study. Am. J. Obstet. Gynecol. 217, 67.e1-67.e21 (2017).

53.Harris, L. K. IFPA Gabor Than Award lecture: Transformation of the spiral arteries in human pregnancy: key events in the remodelling timeline. Placenta 32 Suppl 2, S154-8 (2011).

54.Labarrere, C. A. et al. Failure of physiologic transformation of spiral arteries, endothelial and trophoblast cell activation, and acute atherosis in the basal plate of the placenta. Am. J. Obstet. Gynecol. 216, 287.e1-287.e16 (2017).

55.Yigit, B. et al. SLAMF6 as a regulator of exhausted CD8+ T cells in cancer. Cancer Immunol. Res. 7, 1485–1496 (2019).

56.Bandyopadhyay, S. et al. Role of the putative tumor metastasis suppressor gene Drg-1 in breast cancer progression. Oncogene 23, 5675–5681 (2004).

57.Bandyopadhyay, S. et al. The Drg-1 gene suppresses tumor metastasis in prostate cancer. Cancer Res. 63, 1731–1736 (2003).

58.Kim, N. et al. Comprehensive Analysis for Anti-Cancer Target-Indication Prioritization of Placental Growth Factor Inhibitor (PGF) by Use of Omics and Patient Survival Data. Biology (Basel) 12, (2023).

59.Agca, S. & Kir, S. EDA2R-NIK signaling in cancer cachexia. Curr. Opin. Support. Palliat. Care (2024) doi:10.1097/SPC.0000000000000705.

60.Ge, S. et al. Identification of a Costimulatory Molecule-Related Signature for Predicting Prognostic Risk in Prostate Cancer. Front. Genet. 12, 666300 (2021).

61.Tang, C., Qin, L. & Li, J. A novel anoikis-related gene signature predicts prognosis in patients with breast cancer and reveals immune infiltration. Medicine (Baltimore) 102, e35732 (2023).

62.Baek, S. J. & Eling, T. Growth differentiation factor 15 (GDF15): A survival protein with therapeutic potential in metabolic diseases. Pharmacol. Ther. 198, 46–58 (2019).

63.Wallentin, L. et al. GDF-15 for prognostication of cardiovascular and cancer morbidity and mortality in men. PLoS ONE 8, e78797 (2013).

64.Gebauer, F. et al. Carcinoembryonic antigen-related cell adhesion molecules (CEACAM) 1, 5 and 6 as biomarkers in pancreatic cancer. PLoS ONE 9, e113023 (2014).

65.Zhang, X., Han, X., Zuo, P., Zhang, X. & Xu, H. CEACAM5 stimulates the progression of non-small-cell lung cancer by promoting cell proliferation and migration. J. Int. Med. Res. 48, 300060520959478 (2020).

66.Messaritakis, I. et al. Prognostic significance of CEACAM5mRNA-positive circulating tumor cells in patients with metastatic colorectal cancer. Cancer Chemother. Pharmacol. 82, 767–775 (2018).

67.Saha, S. K., Kim, K., Yang, G.-M., Choi, H. Y. & Cho, S.-G. Cytokeratin 19 (KRT19) has a Role in the Reprogramming of Cancer Stem Cell-Like Cells to Less Aggressive and More Drug-Sensitive Cells. Int. J. Mol. Sci. 19, (2018).

68.Wang, X.-M., Zhang, Z., Pan, L.-H., Cao, X.-C. & Xiao, C. KRT19 and CEACAM5 mRNA-marked circulated tumor cells indicate unfavorable prognosis of breast cancer patients. Breast Cancer Res. Treat. 174, 375–385 (2019).

69.Mori, J. O. et al. Molecular and pathological subtypes related to prostate cancer disparities and disease outcomes in African American and European American patients. Front. Oncol. 12, 928357 (2022).

70.Guo, S. et al. The Role and Therapeutic Value of Syndecan-1 in Cancer Metastasis and Drug Resistance. Front. Cell Dev. Biol. 9, 784983 (2021).
