# Supplementary material for: A machine learning approach to leveraging electronic health records for enhanced omics analysis
Source: Nat Mach Intell. 2025 Jan 16;7(2):293–306. doi: 10.1038/s42256-024-00974-9 (PMC11847705; doi:10.1038/s42256-024-00974-9)
Supplement: Supplementary file 2 — Reporting Summary [file 42256_2024_974_MOESM2_ESM.pdf]

nature portfolio

Corresponding author(s): Nima Aghaeepour, PhD

Last updated by author(s): Dec 4, 2024

Reporting Summary

Nature Portfolio wishes to improve the reproducibility of the work that we publish. This form provides structure for consistency and transparency in reporting. For further information on Nature Portfolio policies, see our Editorial Policies and the Editorial Policy Checklist.

Statistics

For all statistical analyses, confirm that the following items are present in the figure legend, table legend, main text, or Methods section.

n/a	Confirmed	
☐	☒	The exact sample size (*n*) for each experimental group/condition, given as a discrete number and unit of measurement
☐	☒	A statement on whether measurements were taken from distinct samples or whether the same sample was measured repeatedly
☐	☒	The statistical test(s) used AND whether they are one- or two-sided<br>*Only common tests should be described solely by name; describe more complex techniques in the Methods section.*
☐	☒	A description of all covariates tested
☐	☒	A description of any assumptions or corrections, such as tests of normality and adjustment for multiple comparisons
☐	☒	A full description of the statistical parameters including central tendency (e.g. means) or other basic estimates (e.g. regression coefficient) AND variation (e.g. standard deviation) or associated estimates of uncertainty (e.g. confidence intervals)
☐	☒	For null hypothesis testing, the test statistic (e.g. *F*, *t*, *r*) with confidence intervals, effect sizes, degrees of freedom and *P* value noted<br>*Give P values as exact values whenever suitable.*
☒	☐	For Bayesian analysis, information on the choice of priors and Markov chain Monte Carlo settings
☒	☐	For hierarchical and complex designs, identification of the appropriate level for tests and full reporting of outcomes
☐	☒	Estimates of effect sizes (e.g. Cohen's *d*, Pearson's *r*), indicating how they were calculated

Our web collection on statistics for biologists contains articles on many of the points above.

Software and code

Policy information about availability of computer code

Data collection	No new data was collected for this study. No software was used to collect data for this study.
Data analysis	Code can be found at https://github.com/samson920/COMET<br>The following packages and versions are relevant to our analysis:<br>Python: 3.10.6<br>NumPy: 1.23.3<br>Pandas: 1.5.0<br>SciPy: 1.9.1<br>scikit-learn: 1.1.2<br>PyTorch: 1.12.1<br>Gensim: 4.3.0

For manuscripts utilizing custom algorithms or software that are central to the research but not yet described in published literature, software must be made available to editors and reviewers. We strongly encourage code deposition in a community repository (e.g. GitHub). See the Nature Portfolio guidelines for submitting code & software for further information.

Data

Policy information about availability of data

All manuscripts must include a data availability statement. This statement should provide the following information, where applicable:

- Accession codes, unique identifiers, or web links for publicly available datasets
- A description of any restrictions on data availability
- For clinical datasets or third party data, please ensure that the statement adheres to our policy

The proteomics data for the pregnancy cohort are available at Dryad (http://datadryad.org/ and https://doi.org/10.5061/dryad.280gb5mpd). The EHR data for the pregnancy cohort is not able to be shared publicly due to Stanford policies. The data (both proteomics and EHR) for the cancer mortality cohort are available through UK Biobank but cannot be shared publicly due to UK Biobank's data use policies. The queries to pull the cohorts used in our study are included in the code at the GitHub link below, and approved researchers with access to UK Biobank can replicate our analyses using these notebooks and the GitHub tutorial.

The dataset used to externally validate the onset of labor feature importance can be found here: https://nalab.stanford.edu/multiomicsmulticohortpreterm/. The dataset used to externally validate the cancer mortality feature importance can be found in the supplementary data of the original publication in [40].

Human research participants

Policy information about studies involving human research participants and Sex and Gender in Research.

Reporting on sex and gender	There are two populations in our study. The first population consists of people who delivered babies at Stanford. All participants are female sex, and the findings only apply to those of the female sex. The second population is cancer patients from UK Biobank, and includes both men and women. For both cohorts, their demographics are determined based on EHR data and are included as supplementary tables.
Population characteristics	The details of the two populations (including age, race, and ethnicity) are included as supplementary tables in the manuscript. The mean age at delivery in the pregnancy cohort is 32. The mean age in the cancer cohort is 62.
Recruitment	Our study uses COMET to re-analyze existing datasets, and no additional patients were recruited as part of our study.
Ethics oversight	The Stanford IRB approved the use of the Stanford data.<br><br>UK Biobank has approval from the North West Multi-centre Research Ethics Committee (MREC) as a Research Tissue Bank (RTB) approval. This approval means that researchers do not require separate ethical clearance and can operate under the RTB approval. This RTB approval was granted initially in 2011 and it is renewal on a 5-yearly cycle: hence UK Biobank successfully applied to renew it in 2016 and 2021.

Note that full information on the approval of the study protocol must also be provided in the manuscript.

Field-specific reporting

Please select the one below that is the best fit for your research. If you are not sure, read the appropriate sections before making your selection.

☒ Life sciences ☐ Behavioural & social sciences ☐ Ecological, evolutionary & environmental sciences

For a reference copy of the document with all sections, see nature.com/documents/nr-reporting-summary-flat.pdf

Life sciences study design

All studies must disclose on these points even when the disclosure is negative.

Sample size	No sample size calculation was performed prior to the study. Sample size was determined the number of patients with available omics data in each cohort, and the number of patients with sufficient EHR data for the populations of interest (either women who delivered babies at Stanford or patients with cancer diagnoses in the UK Biobank).
Data exclusions	Data in the EHR which could not be linked to an OMOP concept_id were excluded. These data are represented in OMOP tables with a concept_id of 0, indicating that no matching concept could be found. This exclusion is necessary to ensure standardization and consistency in the data.
Replication	The study findings were replicated in a hold-out validation set comprising 15% of the study individuals, across 25 bootstrapping iterations using a different train/test/validation split. Furthermore, biological discoveries were validated in external, publicly available datasets.
Randomization	Randomization was not performed as it is not possible in studies that utilize observational data such as ours. Participants were randomly allocated to training, testing, and validation sets.

Blinding	Blinding was not applicable to the study design with regards to intervention and outcome as the study design does not contain an intervention. A form of blinding was done by creating the hold-out validation set as it was selected at random.

Reporting for specific materials, systems and methods

We require information from authors about some types of materials, experimental systems and methods used in many studies. Here, indicate whether each material, system or method listed is relevant to your study. If you are not sure if a list item applies to your research, read the appropriate section before selecting a response.

Materials & experimental systems

n/a	Involved in the study
☒	☐ Antibodies
☒	☐ Eukaryotic cell lines
☒	☐ Palaeontology and archaeology
☒	☐ Animals and other organisms
☒	☐ Clinical data
☒	☐ Dual use research of concern

Methods

n/a	Involved in the study
☒	☐ ChIP-seq
☒	☐ Flow cytometry
☒	☐ MRI-based neuroimaging
